# 3D and 2D aromatic units behave like oil and water in the case of benzocarborane derivatives

**DOI:** 10.1038/s41467-022-31267-7

**Published:** 2022-07-04

**Authors:** Jordi Poater, Clara Viñas, Miquel Solà, Francesc Teixidor

**Affiliations:** 1 Departament de Química Inorgànica i Orgànica & Institut de Química Teòrica i Computacional (IQTCUB), Universitat de Barcelona, Martí i Franquès 1-11, 08028 Barcelona, Spain; 2 ICREA, Pg. Lluís Companys 23, 08010 Barcelona, Spain; 3 Institut de Ciència de Materials de Barcelona, Consejo Superior de Investigaciones Científicas, Campus Universitat Autònoma de Barcelona, 08193 Bellaterra, Spain; 4 Institut de Química Computacional i Catàlisi and Departament de Química, Universitat de Girona, C/ Maria Aurèlia Capmany, 69, 17003 Girona, Catalonia Spain

**Keywords:** Computational chemistry, Chemical bonding, Density functional theory

## Abstract

A large number of 2D/2D and 3D/3D aromatic fusions that keep their aromaticity in the fused compounds have been synthesized. In addition, we have previously proven the electronic relationship between the 3D aromaticity of boron hydrides and the 2D aromaticity of PAHs. Here we report the possible existence of 3D/2D aromatic fusions that retain the whole aromaticity of the two units. Our conclusion is that such a 3D/2D aromatic combination is not possible due to the ineffective overlap between the π-MOs of the planar species and the n + 1 molecular orbitals in the aromatic cage that deter an effective electronic delocalization between the two fused units. We have also proven the necessary conditions for 3D/3D fusions to take place, and how aromaticity of each unit is decreased in 2D/2D and 3D/3D fusions.

## Abstract (web summary)

2D/2D fusion of aromatic halves leading to a global aromatic is found in many polycyclic aromatic hydrocarbons, whereas 2D/3D aromaticity is difficult to achieve. Here the authors report a computational chemistry investigation showing that 3D/2D aromatic combination is not possible.

## Introduction

The two-dimensional (2D) aromaticity concept is widely accepted in all areas of science, and its most representative example is benzene. In 1978, Aihara^1^ introduced the concept of 3D aromaticity, with the *closo* dodecaborate anion, [B_12_H_12_]^2−^, as its maximum exponent^2^. Very recently, the concept of 3D aromaticity has been revisited. It has been established that a 3D aromatic compound must have a closed-shell electronic structure with at least triply degenerate molecular orbitals (MOs), extensive electron delocalization, and similar magnetic and electronic properties in the three *xyz* directions^3^.

In 2014, an electronic relationship between 2D and 3D aromaticities was reported^4–7^. Conceptually, the 2D aromaticity can be easily extended with the fusion of two 2D aromatic units by sharing one edge. An example is the fusion of two benzenes sharing a common edge that gives rise to a new aromatic compound, naphthalene, and that following the same approach of sharing edges leads to polycyclic aromatic hydrocarbons (PAHs)^8^ such as anthracene, pentacene, perylene, coronene, all the way to graphene, the wonder material of the twenty-first century^9,10^. PAHs are a class of chemicals that are found naturally in coal, crude oil, and gasoline^11^, but they are even found when burning any carbonaceous material^12,13^, or even when cooking meat and other foods at high temperature^14^, or in the interstellar space^15^. The ease with which they are produced is a consequence of the great stability that they have, largely due to the resonance property^16^.

The high stability of 2D aromatic compounds is what favors their great diversity and the fact that they are found in key molecules for life such as hemoglobin, chlorophyll, and DNA bases. Indeed, there is an estimate that, according to PubChem, about two-thirds out of its 110 million structures of chemical compounds are fully or partially aromatic^17^.

Just as there are so many experimental examples of PAHs resulting from the fusion of two 2D aromatic species, which we call 2D/2D for short, showing that the result is another aromatic 2D species, the question is: is the result of a fusion of two 3D/3D aromatic species another aromatic 3D species? And in line to the former one, we also raised the issue of whether it is possible that the fusion of two aromatic species, one 3D and the second 2D, a 3D/2D case, could result in a globally 3D/2D aromatic species.

In this study, we will work as much as possible either with existing molecules or with their derivatives to provide a more consistent basis for the conclusions reached. Contrary to the 2D/2D fusion, there are very few examples available to demonstrate that the fusion of two 3D/3D aromatic entities leads to a molecule with 3D aromaticity. The reason for this is the lack of synthetic methods transferable from one process to another. However, two experimental examples have been found that will prove how the 3D/3D fusion results in a molecule with 3D aromaticity. These studies are based on boron hydride clusters, which are then expanded to hypothetical molecules with the same kind of structure based on existing 3D entities. It is interesting to note that in all known examples of fusions, whether 2D/2D or 3D/3D, the connecting atoms from the building blocks always had two outer electrons from a covalent bond, e.g., C–H or B–H. With this in mind, we have also explored units from polyanions derivatives of Zintl phase compounds^18,19^, e.g., [Sn_12_]^2−^, and fuse them together. The fusion results stress the importance that the connecting atom has one or two exocluster electrons, e.g. C in 2D aromatics or B in 3D aromatics have one electron, whereas Sn has two.

We are aware of the recent synthesis of several fully π-conjugated macrocycles with strongly puckered or cage-type structures that have been considered 3D aromatic^20,21^. However, some authors consider that they are not truly 3D aromatic and rather they should be labeled as 2D-aromatic-in-3D^3^. Therefore, they cannot be used to analyze potential fused systems with 3D/3D aromaticity.

Regarding the question whether the fusion of two entities, a 3D aromatic entity and a 2D aromatic entity, the 3D/2D case, results in a molecule with 3D/2D aromaticity, we will rely on experimental cases which have led to the fusion and demonstrate that in these circumstances the 3D entity retains its aromaticity but the 2D entity loses it. These examples have subsequently been extended to similar but hypothetical molecules that have corroborated these conclusions.

## Results

### 3D/3D fusions

**Fig. 1:**
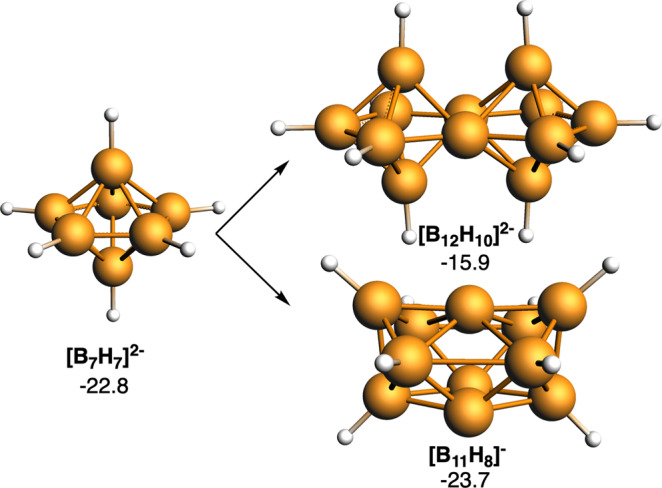
3D/3D systems formed from [B_7_H_7_]^2−^. Computed NICS (in ppm) at the center of the five-membered ring of each cluster are enclosed. Boron atoms in orange and H atoms in white.

In 2014, we published that the electronic equivalent of benzene in boron hydrides was not [B_12_H_12_]^2−^ but [B_7_H_7_]^2−^, i.e., both C_6_H_6_ and [B_7_H_7_]^2−^ share the same number of valence electrons^5^. Therefore, when fusing two benzene building blocks to give a naphthalene, the electronic equivalent in boron hydrides was [B_12_H_10_]^2−^, where two *closo*-B_7_ units share an edge. This compound has not been synthesized but would most likely be stable if there were a pathway for its synthesis, as evidenced by its aromaticity, evaluated through the magnetic-based aromaticity criterion nucleus-independent chemical shift^4–6,22,23^ (NICS—it is considered that the more negative the NICS value of a ring or cage, the higher its aromaticity—Fig. 1). Nonetheless, not in all cases more aromatic is related to more stable, as recently also proven by ourselves in related compounds^23^. If we stick only to the fusion of two [B_7_H_7_]^2−^ units, there would be another *closo* alternative, which would be to share a face instead of an edge leading to [B_11_H_8_]^−^. The pioneering work of Jemmis and other scientists^24^ on a unified rule for predicting and systematizing structures of macropolyhedral boranes has really helped us in our analysis. As can be seen from the NICS shown in Fig. 1, this second option would give rise to a species with a slightly more accentuated aromaticity than the previous one. [B_11_H_8_]^−^ has neither been synthesized, but its higher aromaticity seems to suggest that sharing a face provides more stability than sharing an edge. It is worth noting that [B_11_H_8_]^−^ with *m* = 2 and *n* = 11 and [B_12_H_10_]^2-^ with *m* = 2 and *n* = 12 follow Jemmis’ *mno* rule of stability for fused boranes. According to this rule, the number of skeletal electron pairs required for a condensed polyhedral borane, carborane, heteroborane, metallaborane, or metallocene cluster to be aromatic is given by *m* + *n* + *o*, where *m* = number of sub-clusters, *n* = number of vertices, and *o* = number of single-vertex shared condensations^24,25^.

**Fig. 2:**
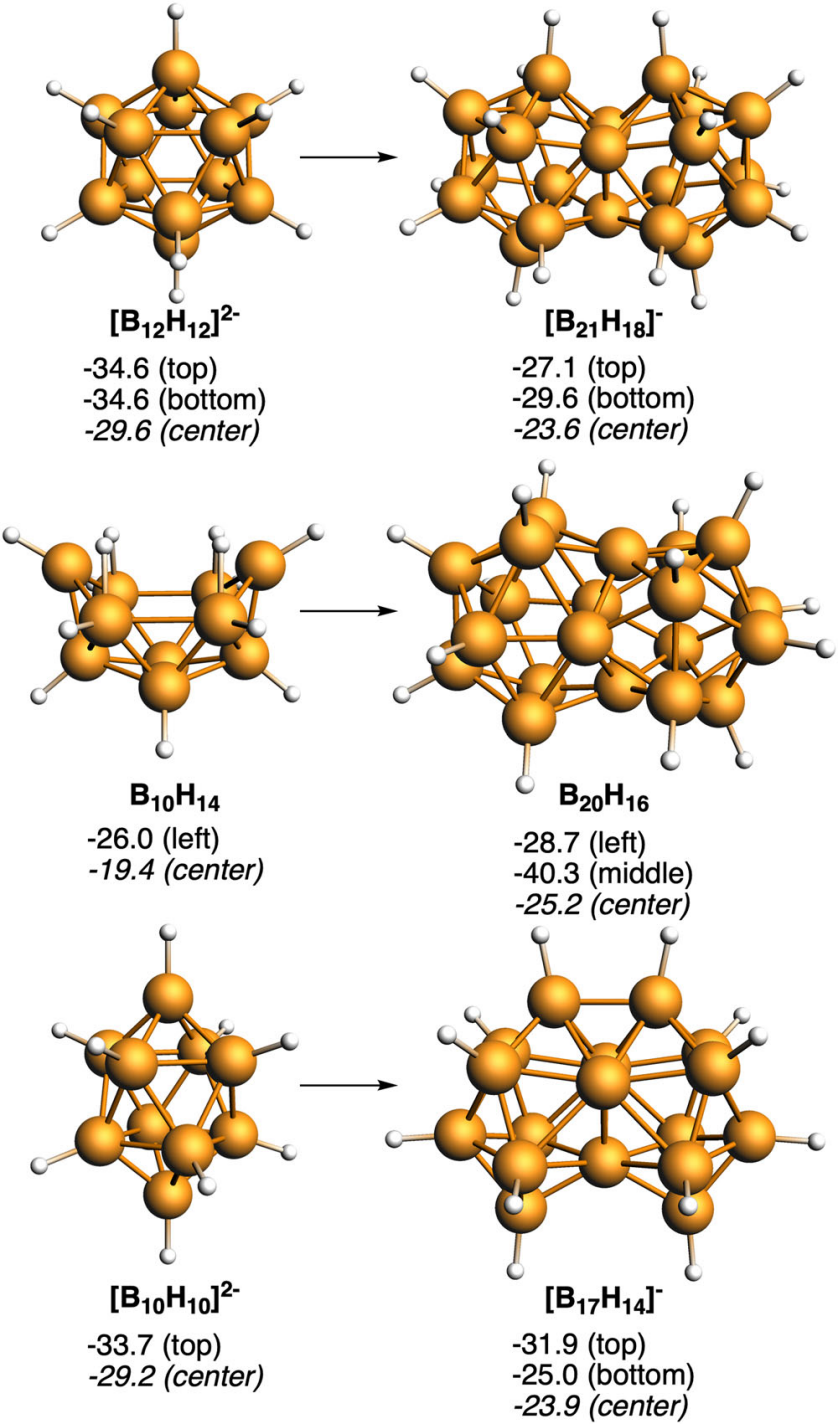
3D/3D systems formed from [B_12_H_12_]^2−^, B_10_H_14_, and [B_10_H_10_]^2−^. Computed NICS (in ppm) at the center of different five- and four-membered rings and at the center of each cluster are enclosed. Boron atoms in orange and H atoms in white.

**Fig. 3:**
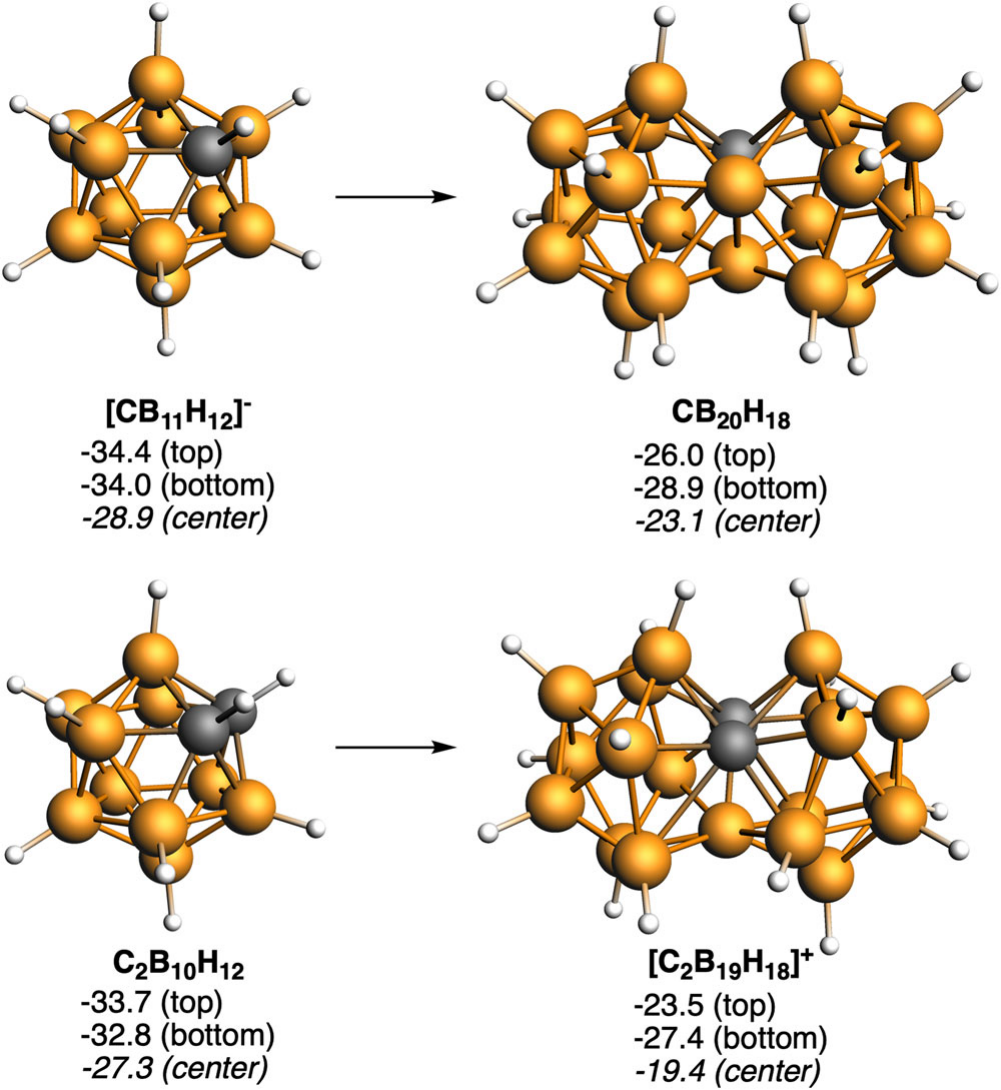
3D/3D systems formed from [CB_11_H_12_]^−^ and C_2_B_10_H_12_. Computed NICS (in ppm) at the center of different five-membered rings and at the center of each cluster are enclosed. Boron atoms in orange, C atoms in gray, and H atoms in white.

These examples are at present imaginary, but are there any synthesized examples that demonstrate the fusion of 3D geometries? The answer is yes. In 2007, Bernhardt, Willner, and coworkers reported the elegant synthesis of [B_21_H_18_]^−^ from *closo*-[B_10_H_10_]^2−^ to *fac*-[B_20_H_18_]^2−^ to *closo*-[B_21_H_18_]^−^, that they named a face-fused diicosahedral borate ion^26^. Earlier, in 1963, two separate groups, those of Lipscomb and Muetterties^27–29^, both using B_10_H_14_ as starting reagent but with distinct synthetic strategies succeeded in synthesizing, isolating, and characterizing B_20_H_16_. Its structure, known by single-crystal determination, is consequence of the B_10_H_14_ boat shape by facing two B_10_H_14_ decks one rotated 90° so as to occupy the minimum volume. Do they share an edge or face? Of the two examples, the *closo*-[B_21_H_18_]^−^ shares one face, three boron atoms producing a global [(BH)_9_B_3_(BH)_9_]^−^, and the B_20_H_16_ can be interpreted as two [B_8_H_8_] units joined by a B_4_ diamond butterfly entity, producing a global [(BH)_8_B_4_(BH)_8_]. Fig. 2 encloses the geometries of these fused compounds and their parent units. In addition, we have also proven the aromaticity, and thus its possible synthetic viability, of [B_17_H_14_]^−^, which would be the result of the fusion of two archetypical species [B_10_H_10_]^2−^. It should be noted that the fusion of two [B_10_H_10_]^2−^ building blocks involves connecting the two apical boron atoms, which are characterized by maintaining their exocluster B–H bonds. This link is also seen later with Sn. And with the aim of completeness, two other boron clusters but based on carboranes, i.e., CB_20_H_18_ and [C_2_B_19_H_18_]^+^, have been also analyzed. These are the fusion products of their also aromatic fusing components, [CB_11_H_12_]^−^ and C_2_B_10_H_12_, respectively (Fig. 3), and were previously proposed by Jemmis et al.^30^, who analyzed their isomerization energies and aromaticity. Here, we have focused only on the isomers in which the C atoms are located on the sharing triangular face derived from either [CB_11_H_12_]^−^ or C_2_B_10_H_12_^30^. We recently reported that the negative NICS values in [B_12_H_12_]^2−^ and C_2_B_10_H_12_ are the result of intense diatropic ring currents inside the cage^23^.

Finally, sandwich metallabis(dicarbollide) compounds (e.g., [Co(C_2_B_9_H_11_)_2_]^−^) can be considered the result of the fusion between two *nido*-[C_2_B_9_H_12_]^−^ cages through a transition metal. In a recent work^23^, we showed that such metallabis(dicarbollide) species display global aromaticity with strong diatropic ring currents inside the two cages. All these boranes and carboranes obey the *mno* rule: [B_21_H_18_]^−^ (*m* = 2, *n* = 21); B_20_H_16_ (*m* = 2, *n* = 20); [B_17_H_14_]^−^ (*m* = 2, *n* = 17); [C_2_B_19_H_18_]^+^ and C_2_B_10_H_12_ (*m* = 2, *n* = 21); and [Co(C_2_B_9_H_11_)_2_]^−^ (*m* = 2, *n* = 23, o = 1)^24^.

**Fig. 4:**
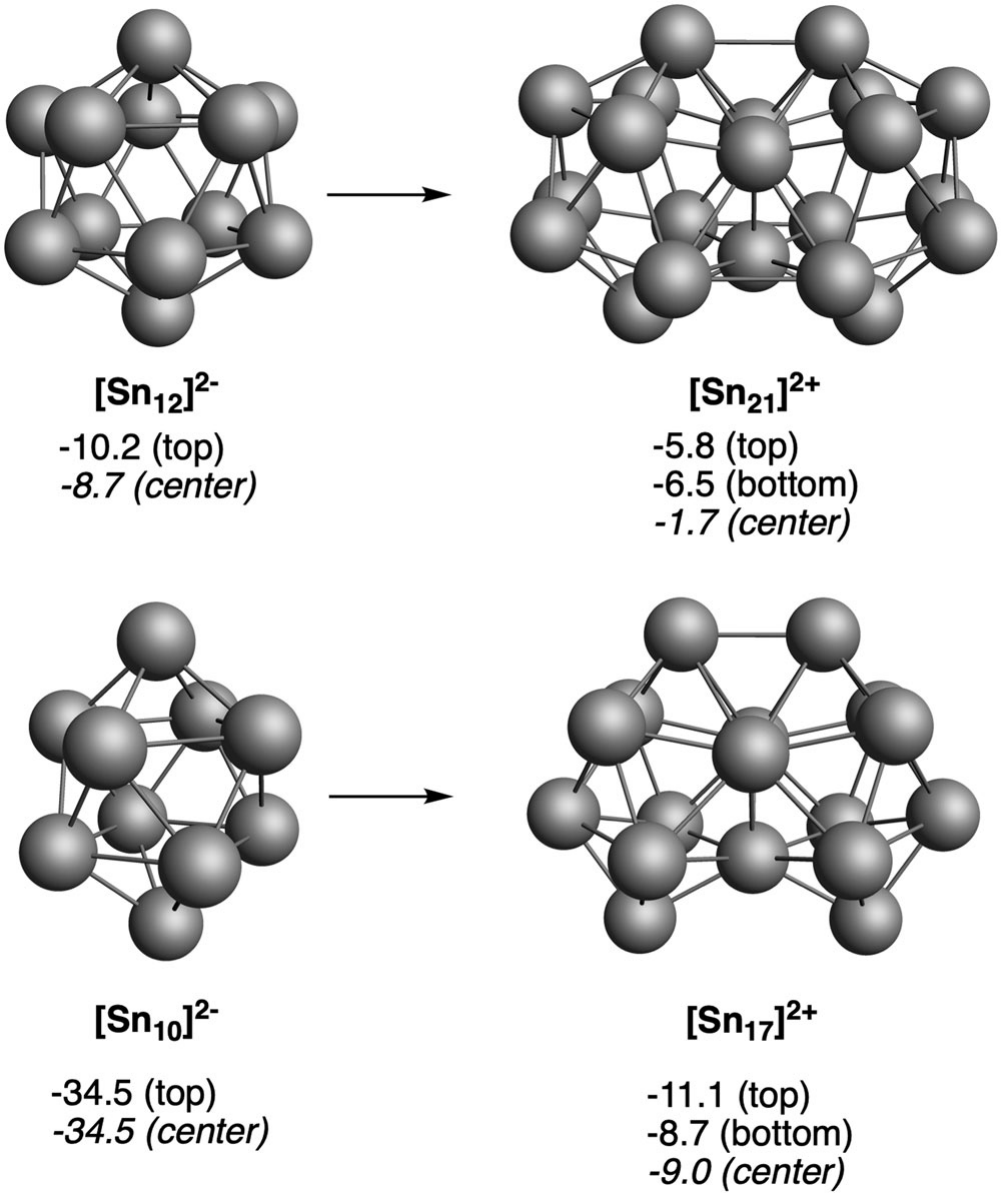
3D/3D systems formed from [Sn_12_]^2−^ and [Sn_10_]^2−^. Computed NICS (in ppm) at the center of different five- and four-membered rings and at the center of each cluster are enclosed. Sn atoms in gray.

Both synthesized *closo*-[B_21_H_18_]^−^ and *closo*-B_20_H_16_ are definitely aromatic as are their fusing components, [B_12_H_12_]^2−^, and B_10_H_14_, respectively. In fact, *closo*-B_20_H_16_ with 22 skeletal electron pairs is aromatic and stable according to Jemmis’ *mno* rule. Remarkable are the connecting elements in fused aromatic hydrocarbons and aromatic *closo* boron hydrides, e.g., C_2_ in naphthalene, (CH)_4_C_2_(CH)_4_ and B_3_ in [(BH)_9_B_3_(BH)_9_]^−^, and B_4_ in [(BH)_8_B_4_(BH)_8_]. The connecting elements do not have any exocluster or exoring substituent when actually their parent components did have one or more exocluster substituents. The issue of the connecting elements is very relevant. For our purposes aimed at demonstrating the global aromaticity of fused 3D aromatic systems, we focus on merging components whose polyhedron is the icosahedron and the bicapped square antiprism. We have remarked that the connecting elements have no substituents outside the ring or cluster. Extending this idea to the elements with lone pairs means that the connecting elements have no electron pairs outside the cluster. In the case of the made [Sn_12_]^2−^ (see ref. ^31^), a *closo* species with the same number of cluster electrons as [B_12_H_12_]^2−^, can be considered^32^. In the same way that the fusion of two [B_12_H_12_]^2−^ gives rise to *closo*-[B_21_H_18_]^−^ one might think that the fusion of two [Sn_12_]^2−^ would give rise to a [Sn_21_]^−^. But that cannot be the case because of the electronic nakedness of the connecting elements. The electronically equivalent compound to *closo*-[B_21_H_18_]^−^ following the *mno* rule would be [Sn_21_]^2+^, shown in Fig. 4. Both compounds *closo*-[B_21_H_18_]^−^ and *closo*-[Sn_21_]^2+^, resulting from 3D/3D fusions, are global aromatic and the same happens with *closo*-[Sn_17_]^2+^, which arises from outstandingly aromatic *closo*-[Sn_10_]^2−^ (Fig. 4), and that can be considered the equivalent to [B_17_H_14_]^−^ and its fusing [B_10_H_10_]^2−^ unit, respectively (Fig. 2), sharing not only the same number of valence electrons but also their aromatic character. Remarkable is that the fused entities display considerably lower NICS values than their corresponding building blocks. Concern could here arise about the absolute interpretation of the NICS values for such heavy atoms like Sn, although it has been recently shown by Foroutan-Nejad et al. that the use of NICS is especially problematic when involving metals with half-filled shells, causing strong local paramagnetic currents. This is not our case, therefore its use in our closed-shell Sn clusters is most probably legitimate^33,34^. However, despite the relatively low negative NICS values found for fused Sn clusters as compared to those of borane clusters, the number of delocalized electrons per B or Sn atom obtained using QTAIM theory^35,36^ of the series of Sn clusters and that of [B_10_H_10_]^2−^ is very similar (Supplementary Fig. 2), thus supporting the aromatic character of both species. Moreover, there is a trend observed in all the reported fusions that is consistent with the aromaticity of fused moieties. The latter have always less negative NICS values than the building blocks. We attribute this reduction in the absolute value of NICS, first, to the intrinsic reduction in the aromaticity due to the fusion and, second, to the coupling between the magnetic fields of the two building blocks in the fused moieties. We can see from the above discussed NICS that the aromaticity of the pairs [B_21_H_18_]^–^/[B_12_H_12_]^2−^, [B_17_H_14_]^–^/[B_10_H_10_]^2−^, CB_20_H_18_/[CB_11_H_12_]^2−^, and [C_2_B_19_H_18_]^+^/C_2_B_10_H_12_ is reduced to a similar extent from the monomer to the dimer. Then, if we consider CB_20_H_18_ and [C_2_B_19_H_18_]^+^^30^, in both cases the connecting elements have carbons, one and two, respectively, or in other words, four or eight electrons are incorporated into the cluster. This enables the charge of the fused species to be more positive. These compounds with the highest atomic electron participation in the cluster turn out to be the fused compounds with the largest decrease from the monomer to the fused cluster. According to this reasoning, what happens with the fused Sn compounds, where there are more connecting elements with a contribution of four electrons each and are larger? As might be expected, the loss of aromaticity is more significant in this case, for both [Sn_21_]^2+^/[Sn_12_]^2−^ and [Sn_17_]^2+^/[Sn_10_]^2−^. This leads to the important conclusion that it is much more difficult to fuse aromatic units of heavier elements than of lighter elements.

**Fig. 5:**
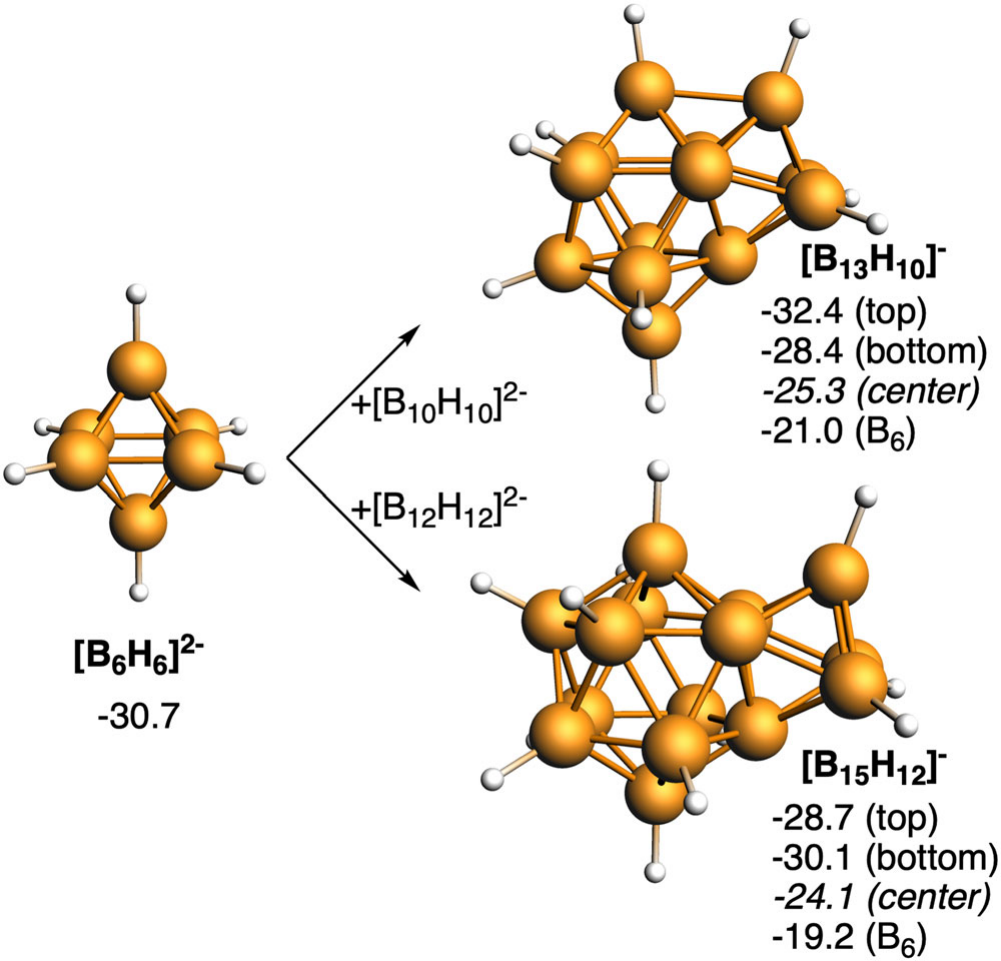
3D/3D systems formed from the fusion of [B_6_H_6_]^2−^ and [B_12_H_12_]^2−^ or [B_10_H_10_]^2−^. Computed NICS (in ppm) at the center of different four- and five-membered rings and at the center of each cluster are enclosed. Boron atoms in orange, and H atoms in white.

To this point, we can state that, for certain types of fusion and electronic charge of the cluster, the fusion of two aromatic halves of light elements results in another aromatic fused species and this stability seems to decrease with increasing atomic weight and the existence of lone pairs in the fusing atom which entail more positive charges in the fusion product, a factor which seems to be destabilizing. On this basis, we have begun with the most advantageous possible situation; we have merged equal halves in shape, size, and composition. For example, two benzenes or two dodecaborates. However, what happens if we fuse two 3D aromatic units of different size? For such, we have attempted the fusion of *closo*-[B_10_H_10_]^2−^ and [B_12_H_12_]^2−^ to smaller [B_6_H_6_]^2−^ to give [B_13_H_10_]^−^ and [B_15_H_12_]^[−38^, respectively, in which both monomers share a 3-membered face (Fig. 5). From the geometries, we can observe that [B_13_H_10_]^−^ also involves connecting the two apical boron atoms, whereas when the difference of size of the two units is larger, like in [B_15_H_12_]^−^, this connection disappears. This latter point agrees with the work by Jemmis et al. stating that a large polyhedral borane condenses preferentially with a smaller polyhedron owing to orbital compatibility^37^. Importantly, the reduction of the aromaticity of the smaller unit ([B_6_H_6_]^2−^) with the fusion is larger (from −30.7 to −21.0/−19.2 ppm) than for either [B_10_H_10_]^2−^ (from −29.2 to −25.3 ppm) or [B_12_H_12_]^2−^ (from −29.6 to −24.1 ppm).

### 3D/2D fusions

And then, what happens if we fuse a 3D aromatic unit with a 2D unit, in which one unit is larger than the second one? To make this situation more propitious, we will make both starting halves visible in the final product. For this purpose, 1,2-C_2_B_10_H_12_ will be used, in which there are two contiguous carbons that can participate in the formation of a 6-carbon ring, a “benzene”. Before studying this case, we would like to recall that [B_12_H_12_]^2−^ represents the archetype of 3D aromaticity, as stated above, and that any compound that can be formally derived from it must, in principle, be less or at the most equally aromatic than the reference compound. It is remarkable that the extraordinary stability of [B_12_H_12_]^2−^ forces 1,2-C_2_B_10_H_12_, albeit incorporating two carbon atoms, to adapt to the geometry and aromaticity of [B_12_H_12_]^2−^ (see ref. ^23^). Therefore, the C···C distance in neutral derivatives of 1,2-C_2_B_10_H_12_ that spans from 1.64 to 1.82 Å^38^, is far from the usual distances in organic compounds approaching the B···B distance in [B_12_H_12_]^2−^ that is ~1.8 Å. Consequently, due to the unequal C–C distance there will be a conflict of stabilities between one aromatic entity ~1.4 Å in benzene and ~1.7 Å in 1,2-C_2_B_10_H_12_. As a result, it may be that the C···C distance reaches an intermediate distance while maintaining a global aromaticity or that only one of the two entities maintains aromaticity, and in this case which of the two? In principle, we could assume that the one that has more to lose is going to be the winner.

**Fig. 6:**
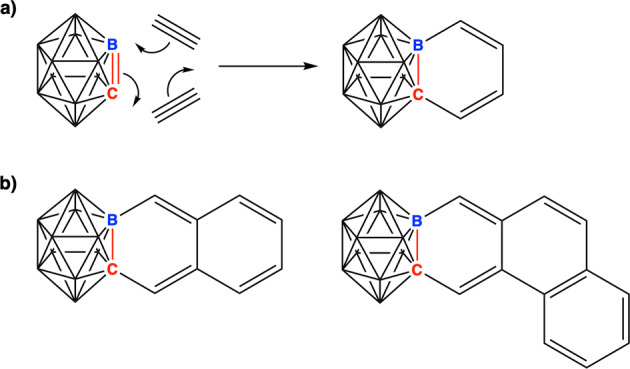
3D + 2D formation. **a** [2 + 2 + 2] cycloaddition between *o*-carboryne and alkynes; **b** structures of fused carborane and PAHS.

**Fig. 7:**
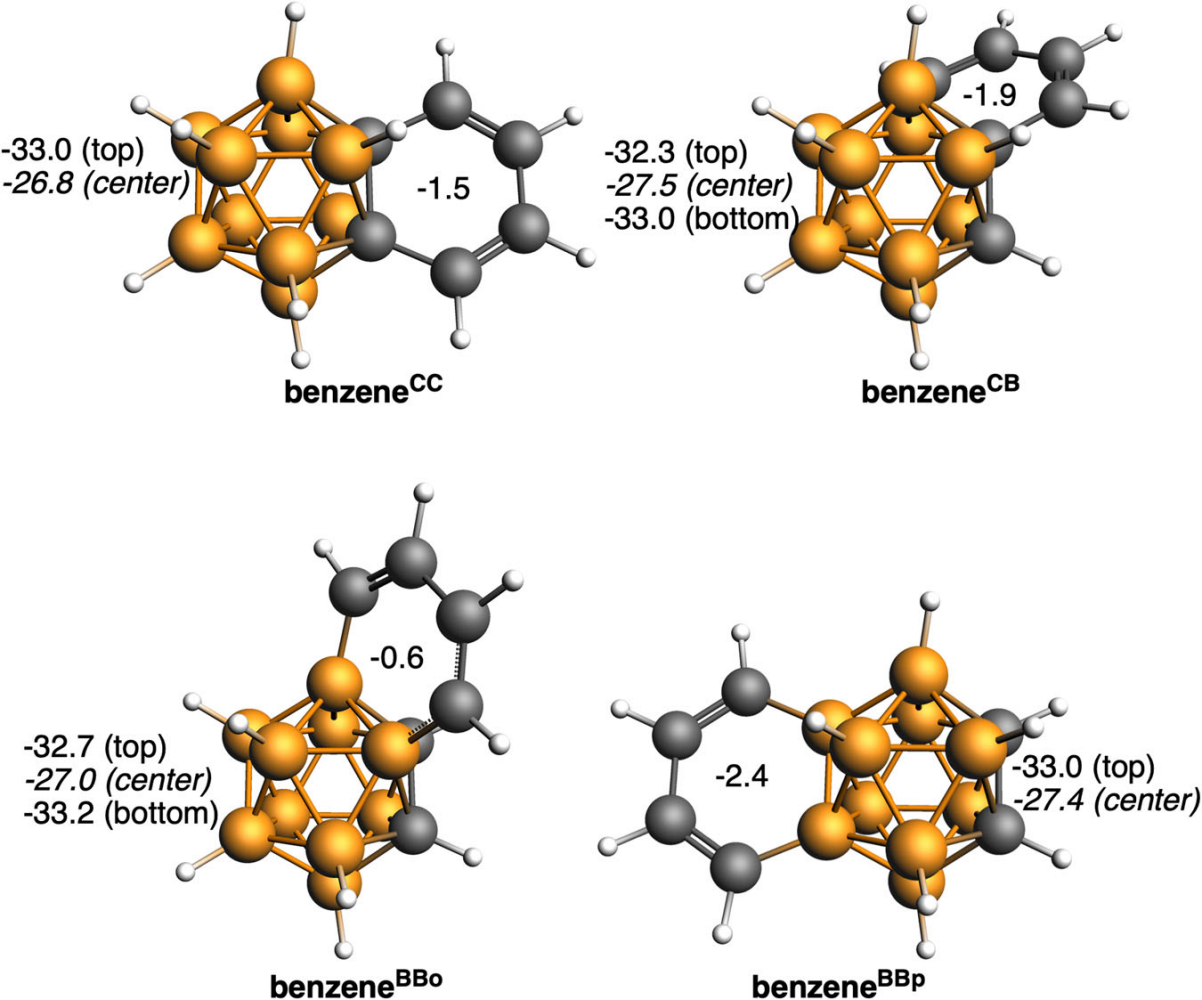
Fused systems between *o*-carborane C_2_B_10_H_12_ and benzene. NICS (in ppm) and nomenclature of the different isomers (BBo states for BB bond next to CC, whereas BBp states for the BB bond at the opposite site to the CC bond). NICS for C_2_B_10_H_12_ are −33.3 and −27.3 ppm for the center of the B_4_C ring and center of the cluster (values in italics), respectively, and −8.1 ppm for benzene (see also Supplementary Tables 1 and 2). Boron atoms in orange, C atoms in gray, and H atoms in white.

The optimal example to be studied is the formal fusion of 1,2-C_2_B_10_H_12_ and benzene. Fortunately, the compound was previously synthesized and is known as the benzocarborane for which different synthetic methods have been described based on either *o*-carborane or carboryne as starting reagents^21,39,40^. Benzocarborane can also be synthesized from a transition metal-catalyzed [2 + 2 + 2] cycloaddition^41^ of carboryne and two acetylene molecules (Fig. 6a)^42^. The name benzocarborane implies that there is a benzene (aromatic) attached to a carborane whose aromatic condition was not indicated. The situation, as we shall see, is quite the opposite. Therefore, benzocarborane and its derivatives (Fig. 6) and its isomers (Fig. 7) are very adequate to address the question whether the 3D/2D aromatic fusion is feasible. Not too many alternatives exist that would facilitate this study. If the 2D fragment is made of carbons, this requires or at least makes it more likely, that the 3D fragment also contains adjacent carbon atoms in the structure, and that both are aromatic. In this context, *o*-carborane, 1,2-C_2_B_10_H_12_ is key to study the 3D/2D fusion^43^. In difference, fullerenes may look appealing but either do not have 3D spherical aromaticity^44^ or, reminding the issue of the connecting elements described earlier, how would it be possible to fuse with another aromatic half without sacrificing (at least partially) the aromaticity of the fullerene? As can be inferred from the above, only two aromatic halves with an outer electron pair in the system, either a C–H or a lone pair, can be fused. It is worth noting that we are not discussing the aromaticity of a phenyl group linked to a carborane, [PhCB_11_H_11_]^−^, as reported recently by Muñoz-Castro (in this case both moieties remain aromatic)^45^ but of benzo-*o*-carborane with the carborane cage and benzene ring fused. Also importantly, Xie and coworkers succeeded in the synthesis and X-ray characterization of substituted carboranes, and proved that there is considerable bond length alternation in the benzene ring (164.8–133.8 pm) ruling out aromaticity^42,46^. Similar alternation bond lengths were proven by Wade and coworkers on pristine benzo-*o*-carborane (165.1–133.8 pm)^47^, in agreement with the ethyl derivative.

**Fig. 8:**
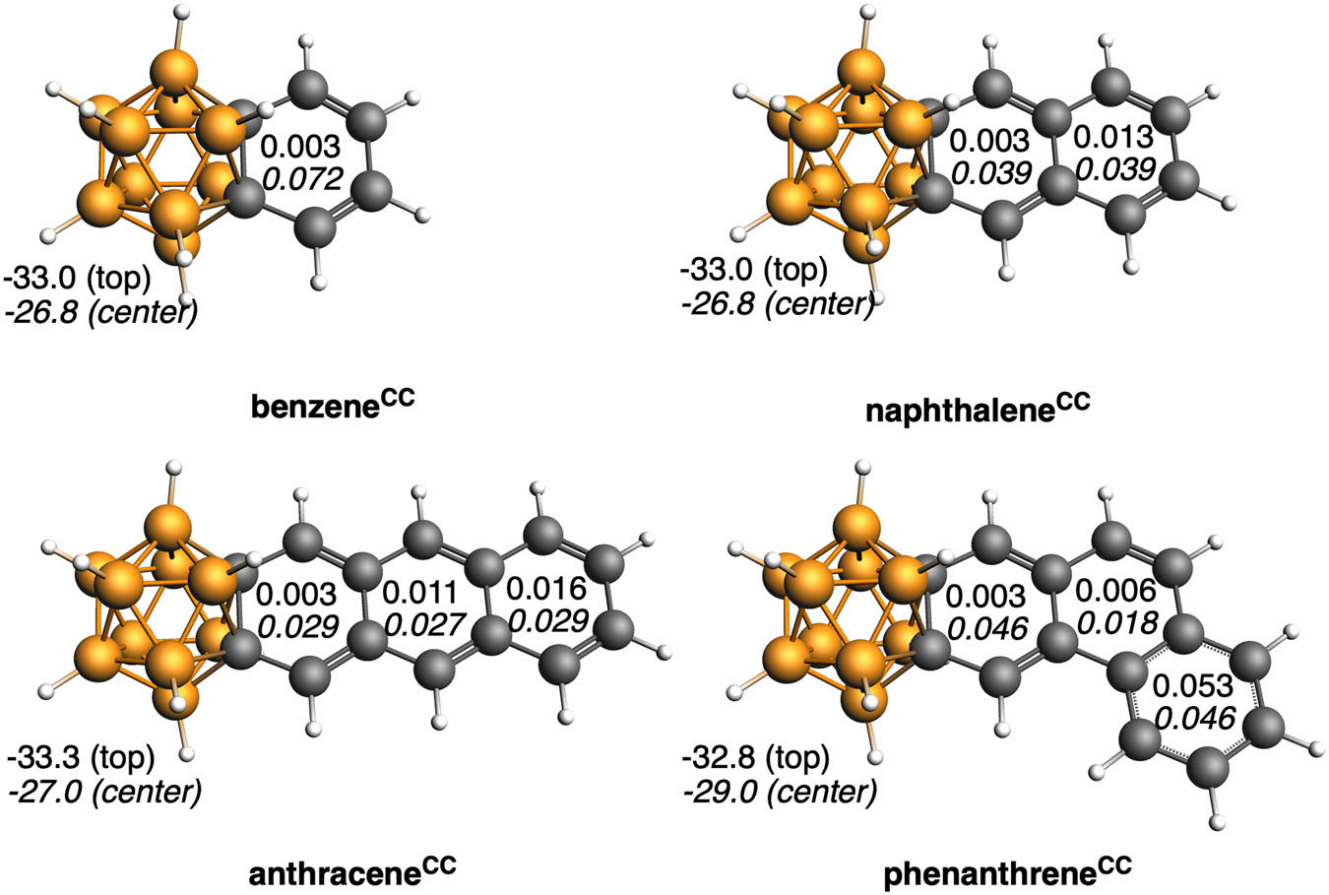
Fused systems between *o*-carborane C_2_B_10_H_12_ and benzene, naphthalene, anthracene, and phenanthrene. NICS (in ppm) for the five-membered rings and center of the carborane and MCI (in au) for the PAHs with and without (in italics) the carborane being attached (see also Supplementary Tables 1 and 2). Boron atoms in orange, C atoms in gray, and H atoms in white. Larger MCI values indicate higher electron delocalization in the ring.

NICS values very close to zero or to positive values indicate non-aromaticity, thus the computed data concerning the 2D fragment in Fig. 7 confirms the Matteson and Hota’s title of their article about benzocarborane, “High stability but little aromatic character”^40^. The NICS value of the 2D fragment is in significant contrast to that of *o*-carborane, which is very negative, indicating that just as the 2D fragment is not very aromatic, the 3D fragment is strongly aromatic. Therefore, of the two fragments, one retains aromaticity while the other clearly loses it. The four benzocarborane isomers studied and presented in Fig. 7 show the same trend, but it is worth noting that the one with the C_4_B_2_ group occupying *para* positions relative to the two carbons of the carborane (benzene^BBp^) has the most negative NICS values. These results point out that the fusion of two halves, each one known to be clearly aromatic, both suitable for the 3D/2D investigation does not lead to a global aromatic compound, but instead one of the two halves loses its aromaticity while the second retains its aromaticity. In the benzocarborane, the carborane keeps its aromaticity, with NICS only slightly reduced (from −33.3 to −33.0 ppm in the center of B_4_C five-membered ring (5-MR) and from −27.3 to −26.8 ppm in the center of cluster), however, the fused benzene becomes nonaromatic (NICS reduced from −8.1 to −1.5 ppm) as expected from the fact that the 6-MR contains only four π-electrons. The same trend is observed when benzene is fused to bonds other than C–C (Fig. 7). And the same behavior applies to larger PAHs: naphthalene, anthracene, and phenanthrene, when fused to *o*-carborane (Fig. 8). The only ring that gives an opposite behavior is the terminal one of phenanthrene. The reason is the presence of a Clar π-sextet^48,49^ on this ring (Fig. 6b), at difference to either naphthalene or anthracene that cannot accommodate such π-sextet (bond lengths also support this observation, Supplementary Fig. 1). This is the reason why only this ring remains fully aromatic.

The difficulty for this 3D/2D fusion can be assigned to the lack of overlap between the π-MOs of the PAH and those of the carborane. A model system built from the system benzene^CC^ above, analyzed through a quantitative molecular orbital and energy decomposition analyses, has allowed to confirm the ineffective overlap (Supplementary Discussion). Finally, let us mention that the above conclusions on 3D/2D fusion also apply to the fused carborane and five-membered heterocycles previously synthesized in ref. ^50^.

## Discussion

One of the fundamental characteristics of Hückel’s 2D aromaticity is the large number of aromatic species derived from the fusion of two aromatic halves. We have named these as 2D/2D aromatic fusions. Prior to this work, few 3D/3D aromatic fusions had been explicitly described^29^, although some examples had been synthesized and have been demonstrated in this work to be global aromatics. It has therefore been shown that such fusions are possible by the union of two 3D aromatic halves. The difficulty or impossibility for 3D/2D fusions is also proven in this work. The main reason for non-having a 3D/2D aromatic species is the ineffective overlap between the π-MOs of the PAH and the *n* + 1 MOs in the cage that deter a higher electronic delocalization between the two fused units.

Furthermore, it is proven that for 3D/3D fusions to take place, some conditions have to be fulfilled. The first and obvious one is that each of the halves to be merged must be aromatic. The halves to be fused must have a great similarity in the two-connecting atom distances, although in borane clusters, 3D units of different size favor fusion^37^. The participating elements in the fusion must have exocluster bonds, e.g., C–H, B–H in the starting half or lone pairs. These connecting elements will be distinct in the fused molecule from other participating elements and will not have exocluster bonds. To our understanding, these conditions appear to be necessary for fusions of aromatic species to give rise to a global aromatic, but may not be sufficient.

It seems that it can be generalized that aromaticity decays with fusion, this is observed in all 2D/2D, 3D/3D, and 3D/2D cases, being in the latter case dramatic as the 2D aromaticity vanishes completely. It is worth noting that the aromaticity decays comparably from 2D to 2D/2D as from 3D to 3D/3D. Fusion is more feasible with elements of the second period, C and B, and probably as one moves down the periodic table the aromaticity decreases. This has been seen with Sn in period 5, although it is in the same group as C. To be a connecting element, the atom in question must have an exocluster or exoring electron pair, e.g., C–H, B–H or Sn. The existence of lone pairs, however, leads to cationic charged species which can be potentially destabilizing.

## Methods

All calculations were performed with the Gaussian 09 package^51^ by means of the B3LYP^52–54^ hybrid density functional and the 6–311 + +G(d,p) basis set^55^. The geometry optimizations were carried out without symmetry constraints (Supplementary Data 1). Analytical Hessians were computed to characterize the optimized structures as minima (zero imaginary frequencies). Sn cluster have been computed by means of AMS 2021 software at ZORA-B3LYP-D3BJ/TZ2P level^56,57^. Aromaticity was evaluated by means of the nucleus-independent chemical shift (NICS)^4–6,22,23^, proposed by Schleyer and coworkers as a magnetic descriptor of aromaticity. NICS is defined as the negative value of the absolute shielding computed at a ring center or at some other point of the system. Rings with large negative NICS values are considered aromatic. NICS values were computed using the gauge-including atomic orbital method (GIAO)^58^. Multicenter indices (MCI)^59–61^ and delocalization indices (DI)^35,36^ were computed with the ESI-3D program using AIM partition of space^62,63^. For completeness, the aromaticity of the enclosed systems has been further confirmed by means of bond length alternation (BLA) measures (Supplementary Table 3).

## Supplementary information

Supplementary Information

Description of Additional Supplementary Files

Supplementary Data 1

## Data Availability

The authors declare that the data supporting the findings of this study are available within the paper and  Supplementary Information and Supplementary Data files.

## References

[1] Aihara J (1978). Three-dimensional aromaticity of polyhedral boranes. J. Am. Chem. Soc..

[2] Pitochelli AR, Hawthorne MF (1960). The Isolation of the Icosahedral B_12_H_12_^-2^ Ion. J. Am. Chem. Soc..

[3] Bakouri OES (2022). Fully π-conjugated macrocycles: when 3D-aromatic and when 2D-aromatic-in-3D?. J. Am. Chem. Soc..

[4] Poater J, Solà M, Viñas C, Teixidor F (2013). A simple link between hydrocarbon and borohydride chemistries. Chem. Eur. J..

[5] Poater J, Solà M, Viñas C, Teixidor F (2014). *π*-Aromaticity and three-dimensional aromaticity: two sides of the same coin?. Angew. Chem. Int. Ed..

[6] Poater J, Solà M, Viñas C, Teixidor F (2016). Hückel’s rule of aromaticity categorizes aromatic *closo* boron hydride clusters. Chem. Eur. J..

[7] Poater, J., Viñas, C., Solà, M. & Teixidor, F. Aromaticity and extrusion of benzenoids linked to [o-COSAN]^−^. Clar has the Answer. *Angew Chem. Int. Ed.* **61**, e202200672 (2022). 10.1002/anie.202200672 PMC9310775 35176201

[8] Patel AB, Shaikh S, Jain KR, Desai C, Madamwar D (2020). Polycyclic aromatic hydrocarbons: sources, toxicity, and remediation approaches. Front. Microbiol..

[9] Geim AK, Novoselov KS (2007). The rise of graphene. Nat. Mater..

[10] Novoselov KS (2004). Electric field effect in atomically thin carbon films. Science.

[11] Abdel-Shafy HI, Mansour MSM (2016). A review on polycyclic aromatic hydrocarbons: Source, environmental impact, effect on human health and remediation. Egypt J. Pet..

[12] Mandalakis M (2005). Contribution of biomass burning to atmospheric polycyclic aromatic hydrocarbons at three European background sites. Environ. Sci. Technol..

[13] Munzel T (2020). Effects of tobacco cigarettes, e-cigarettes, and waterpipe smoking on endothelial function and clinical outcomes. Eur. Heart J..

[14] Falcó G (2003). Polycyclic aromatic hydrocarbons in foods: Human exposure through the diet in Catalonia, Spain. J. Food Prot..

[15] Allamandola LJ, Tielens A, Barker JR (1989). Interstellar polycyclic aromatic-hydrocarbons—the infrared-emission bands, the excitation emission mechanism, and the astrophysical implications. Astrophys J., Suppl. Ser..

[16] Randić M (2003). Aromaticity of polycyclic conjugated hydrocarbons. Chem. Rev..

[17] Balaban AT, Oniciu DC, Katritzky AR (2004). Aromaticity as a cornerstone of heterocyclic chemistry. Chem. Rev..

[18] Hirsch A, Chen ZF, Jiao HJ (2001). Spherical aromaticity of inorganic cage molecules. Angew. Chem. Int. Ed..

[19] Liu C, Popov IA, Chen Z, Boldyrev AI, Sun Z-M (2018). Aromaticity and antiaromaticity in Zintl clusters. Chem. Eur. J..

[20] Ni Y (2020). 3D global aromaticity in a fully conjugated diradicaloid cage at different oxidation states. Nat. Chem..

[21] Wu S (2022). Hückel- and Baird-type 3D global aromaticity in a fully conjugated molecular cage. Angew. Chem. Int Ed..

[22] Chen ZF, Wannere CS, Corminboeuf C, Puchta R, Schleyer PVR (2005). Nucleus-independent chemical shifts (NICS) as an aromaticity criterion. Chem. Rev..

[23] Poater J (2020). Too persistent to give up: aromaticity in boron clusters survives radical structural changes. J. Am. Chem. Soc..

[24] Jemmis ED, Balakrishnarajan MM, Pancharatna PD (2002). Electronic requirements for macropolyhedral boranes. Chem. Rev..

[25] Priyakumari, C. P. & Jemmis, E. D. Electron-counting rules in cluster bonding— polyhedral boranes, elemental- boron, and boron-rich solids. in *The Chemical Bond: Chemical Bonding Across the Periodic Table* (eds Frenking, G. & Shaik, S.) (Wiley-VCH, 2014), pp. 113–147.

[26] Bernhardt E, Brauer DJ, Finze M, Willner H (2007). *closo*-B_21_H_18_^-^: a face-fused diicosahedral borate ion. Angew. Chem. Int. Ed..

[27] Dobrott RD, Friedman LB, Lipscomb WN (1964). Molecular and crystal structure of B_20_H_16_. J. Chem. Phys..

[28] Friedman LB, Dobrott RD, Lipscomb WN (1963). Preparation and structure of a new boron hydride, B_20_H_16_. J. Am. Chem. Soc..

[29] Miller NE, Muetterties EL (1963). A new boron hydride, B_20_H_16_. J. Am. Chem. Soc..

[30] Vidya K, Jemmis ED (2015). Relative stabilities of condensed face sharing mono- and di-carboranes: CB_20_H_18_ and C_2_B_19_H_18_^+^. J. Organomet Chem..

[31] Cui L-F (2006). Sn_12_^2-^: stannaspherene. J. Am. Chem. Soc..

[32] Rudolph RW (1976). Boranes and heteroboranes—paradigm for electron requirements of clusters. Acc. Chem. Res..

[33] Foroutan-Nejad C (2015). Is NICS a reliable aromaticity index for transition metal clusters?. Theor. Chem. Acc..

[34] Cuyacot BJR, Foroutan-Nejad C (2022). [{Th(C_8_H_8_)Cl_2_}_3_]^2−^ is stable but not aromatic. Nature.

[35] Fradera X, Austen MA, Bader RFW (1999). The Lewis model and beyond. J. Phys. Chem. A.

[36] Fradera X, Poater J, Simon S, Duran M, Solà M (2002). Electron-pairing analysis from localization and delocalization indices in the framework of the atoms-in-molecules theory. Theor. Chem. Acc..

[37] Shameema O, Jemmis ED (2009). Relative stability of *closo-closo*, *closo-nido*, and *nido-nido* macropolyhedral boranes: the role of orbital compatibility. Chem. Asian J..

[38] Oliva JM, Allan NL, Schleyer PVR, Viñas C, Teixidor F (2005). Strikingly long C···C distances in 1,2-disubstituted *ortho*-carboranes and their dianions. J. Am. Chem. Soc..

[39] Matteson DS, Grunzinger RE (1974). Benzodicarbollide and dihydrobenzodicarbollide ions and their complexes with manganese, cobalt, and nickel. Inorg. Chem..

[40] Matteson DS, Hota NK (1971). Benzocarborane. High stability but little aromatic character. J. Am. Chem. Soc..

[41] Roglans A, Pla-Quintana A, Solà M (2021). Mechanistic studies of transition-metal-catalyzed [2+2+2] cycloaddition reactions. Chem. Rev..

[42] Deng L, Chan H-S, Xie Z (2006). Nickel-mediated regioselective 2+2+2 cycloaddition of carboryne with alkynes. J. Am. Chem. Soc..

[43] Schleyer PVR, Najafian K (1998). Stability and three-dimensional aromaticity of *closo*-monocarbaborane anions, CB_n-1_H_n_^−^, and *closo*-dicarboranes, C_2_B_n-2_H_n_. Inorg. Chem..

[44] Garcia-Borràs M, Osuna S, Luis JM, Swart M, Solà M (2014). The role of aromaticity in determining the molecular structure and reactivity of (endohedral metallo)fullerenes. Chem. Soc. Rev..

[45] Muñoz-Castro A (2020). Interplay between planar and spherical aromaticity: shielding cone behavior in dual planar-planar, planar-spherical and spherical-spherical aromatics. Chem. Phys. Chem.

[46] Qiu Z, Wang SR, Xie Z (2010). Nickel-catalyzed regioselective [2+2+2] cycloaddition of carboryne with alkynes. Angew. Chem. Int Ed..

[47] Copley, R. C. B. et al. Crystallographic evidence for the diene character of C_2_B_10_H_10_C_4_H_4_ (‘benzocarborane’) and a Diels-Alder reaction of its anionic *nido*-analogue, C_2_B_9_H_10_C_4_H_4_^−^: crystal structures of C_2_B_10_H_10_C_4_H_4_ and C_2_B_10_H_10_C_4_H_6_. *Chem. Comm.* 2033–2034 (1996).

[48] Clar, E. *The Aromatic Sextet* (Wiley, 1972).

[49] Solà M (2013). Forty years of Clar’s aromatic π-sextet rule. Front Chem..

[50] Chan TL, Xie ZW (2018). Synthesis, structure and aromaticity of carborane-fused carbo- and heterocycles. Chem. Sci..

[51] Frisch, M. J. et al. *Gaussian 09* (Gaussian, Inc., 2009).

[52] Becke AD (1993). Density-functional thermochemistry .3. The role of exact exchange. J. Chem. Phys..

[53] Lee CT, Yang WT, Parr RG (1988). Development of the Colle-Salvetti correlation-energy formula into a functional of the electron-density. Phys. Rev. B.

[54] Stephens PJ, Devlin FJ, Chabalowski CF, Frisch MJ (1994). Ab-initio calculation of vibrational absorption and circular-dichroism spectra using density-functional force-fields. J. Phys. Chem..

[55] Frisch MJ, Pople JA, Binkley JS (1984). Self-consistent molecular-orbital methods. 25. Supplementary functions for Gaussian-basis sets. J. Chem. Phys..

[56] SCM AMS 2021.1. *Theoretical Chemistry* (Vrije Universiteit, 2021).

[57] te Velde G (2001). Chemistry with ADF. J. Comput Chem..

[58] Wolinski K, Hinton JF, Pulay P (1990). Efficient implementation of the gauge-independent atomic orbital method for NMR chemical-shift calculations. J. Am. Chem. Soc..

[59] Bultinck P, Ponec R, Van Damme S (2005). Multicenter bond indices as a new measure of aromaticity in polycyclic aromatic hydrocarbons. J. Phys. Org. Chem..

[60] Feixas F, Matito E, Poater J, Solà M (2015). Quantifying aromaticity with electron delocalisation measures. Chem. Soc. Rev..

[61] Matito E, Duran M, Solà M (2005). The aromatic fluctuation index (FLU): a new aromaticity index based on electron delocalization. J. Chem. Phys..

[62] Matito, E. *ESI-3D: Electron Sharing Indices Program for 3D Molecular Space Partitioning, Institute of Computational Chemistry and Catalysis (IQCC)* (University of Girona, 2006).

[63] Matito E, Solà M, Salvador P, Duran M (2007). Electron sharing indexes at the correlated level. Application to aromaticity calculations. Faraday Discuss.

