# Supplementary material for: 3D and 2D aromatic units behave like oil and water in the case of benzocarborane derivatives
Source: Nat Commun. 2022 Jul 4;13:3844. doi: 10.1038/s41467-022-31267-7 (PMC9253344; doi:10.1038/s41467-022-31267-7)
Supplement: Supplementary file 1 — Supplementary Information [file 41467_2022_31267_MOESM1_ESM.pdf]

SUPPLEMENTARY INFORMATION

SUPPLEMENTARY TABLES

Supplementary Table 1. NICS (in ppm) and MCI (in e) of rings of fused carborane and PAH system, and relative energies compared to the most stable isomer (in kcal mol^{-1}).

	NICS carborane			NICS PAH			MCI PAH			
**system**	**B_4C^{top}**	**B_4C^{bott}**	**center**	**C_4X_2**	**C_6^{mid}**	**C_6^{ter}**	**C_4X_2**	**C_6^{mid}**	**C_6^{ter}**	**ΔE**
**benzene^{CC}**	-33.0	-33.0	-26.8	-1.5			0.003			10.85
**benzene^{CB}**	-32.3	-33.0	-27.5	-1.9			0.002			4.62
**benzene^{BBo}**	-32.7	-33.2	-27.0	-0.6			0.002			0.00
**benzene^{BBp}**	-33.0	-33.0	-27.4	-2.4			0.003			1.27
**naphthalene^{CC}**	-33.0	-33.0	-26.8	-2.7	1.0		0.003	0.013		10.40
**naphthalene^{CB}**	-32.8	-33.2	-28.3	-3.6	-0.4		0.002	0.014		3.98
**naphthalene^{BBo}**	-32.7	-33.6	-27.0	-1.8	1.7		0.002	0.013		0.00
**naphthalene^{BBp}**	-32.8	-32.8	-27.3	-4.1	0.5		0.002	0.014		0.69
**anthracene^{CC}**	-33.3	-33.3	-27.0	-3.4	0.1	1.5	0.003	0.011	0.016	10.31
**anthracene^{CB}**	-33.2	-33.5	-28.9	-4.6	-1.0	0.8	0.002	0.011	0.016	3.68
**anthracene^{BBo}**	-34.0	-32.8	-26.9	-2.4	0.9	1.8	0.002	0.011	0.016	0.00
**anthracene^{BBp}**	-32.9	-32.9	-27.5	-5.0	-1.1	0.8	0.002	0.012	0.017	0.35
**phenanthrene^{CC}**	-32.8	-33.2	-29.0	-2.2	2.3	-6.8	0.003	0.006	0.053	9.70
**phenanthrene^{CB}**	-32.6	-33.0	-28.2	-2.9	2.1	-7.0	0.002	0.007	0.053	4.02
**phenanthrene^{BBo}**	-32.7	-32.4	-28.4	-1.0	3.2	-6.7	0.002	0.006	0.054	0.00
**phenanthrene^{BBp}**	-32.7	-33.0	-28.0	-3.2	2.4	-6.9	0.002	0.007	0.053	1.07

Supplementary Table 2. NICS (in ppm) and MCI (in e) of $C_2B_{10}H_{12}$ and reference PAHs.

	NICS			MCI		
**system**	**C_6^{int}**	**C_6^{mid}**	**C_6^{ter}**	**C_6^{int}**	**C_6^{mid}**	**C_6^{ter}**
**benzene**	-8.1			0.072		
**naphthalene**	-8.5	-8.5		0.039	0.039	
**anthracene**	-7.4	-11.4	-7.4	0.029	0.027	0.029
**phenanthrene**	-8.5	-5.6	-8.5	0.046	0.018	0.046
	**B_4C^{top}**	**B_4C^{bott}**	**center**			
**$C_2B_{10}H_{12}$**	-33.3	-33.3	-27.3			

Supplementary Table 3. Bond length alternation (BLA, in Å) for the series of compounds under analysis. BLA has been computed as the longest and shortest bond lengths for each system. In the fused systems, no atom involved in the fusion between the two units has been considered. Only B-B bonds have been considered for the carboranes.

	shortest	*longest*	BLA
$[B_7H_7]^{2-}$	*1.656*	*1.830*	0.174
$[B_{12}H_{10}]^{2-}$	*1.665*	*1.809*	0.144
$[B_{11}H_8]^-$	*1.678*	*1.883*	0.205
$[B_{12}H_{12}]^{2-}$	*1.783*	*1.783*	0.000
$[B_{21}H_8]^-$	*1.735*	*1.803*	0.068
$B_{10}H_{14}$	*1.722*	*1.980*	0.258
$B_{20}H_{16}$	*1.754*	*1.800*	0.046
$[B_{10}H_{10}]^{2-}$	*1.701*	*1.840*	0.139
$[B_{17}H_{14}]$-	*1.685*	*1.938*	0.253
$[CB_{11}H_{12}]$-	*1.772*	*1.785*	0.013
$CB_{20}H_{18}$	*1.700*	*1.829*	0.129
$C_2B_{10}H_{12}$	*1.761*	*1.789*	0.028
$[C_2B_{19}H_{18}]^+$	*1.676*	*1.834*	0.158
$[Sn_{12}]^{2-}$	*3.242*	*3.242*	0.000
$[Sn_{21}]^{2+}$	*3.161*	*3.326*	0.165
$[Sn_{10}]^{2-}$	*3.111*	*3.413*	0.302
$[Sn_{17}]^{2+}$	*3.054*	*3.621*	0.567

SUPPLEMENTARY FIGURES

Supplementary Figure 1 Bond lengths (in Å) of the PAHs.

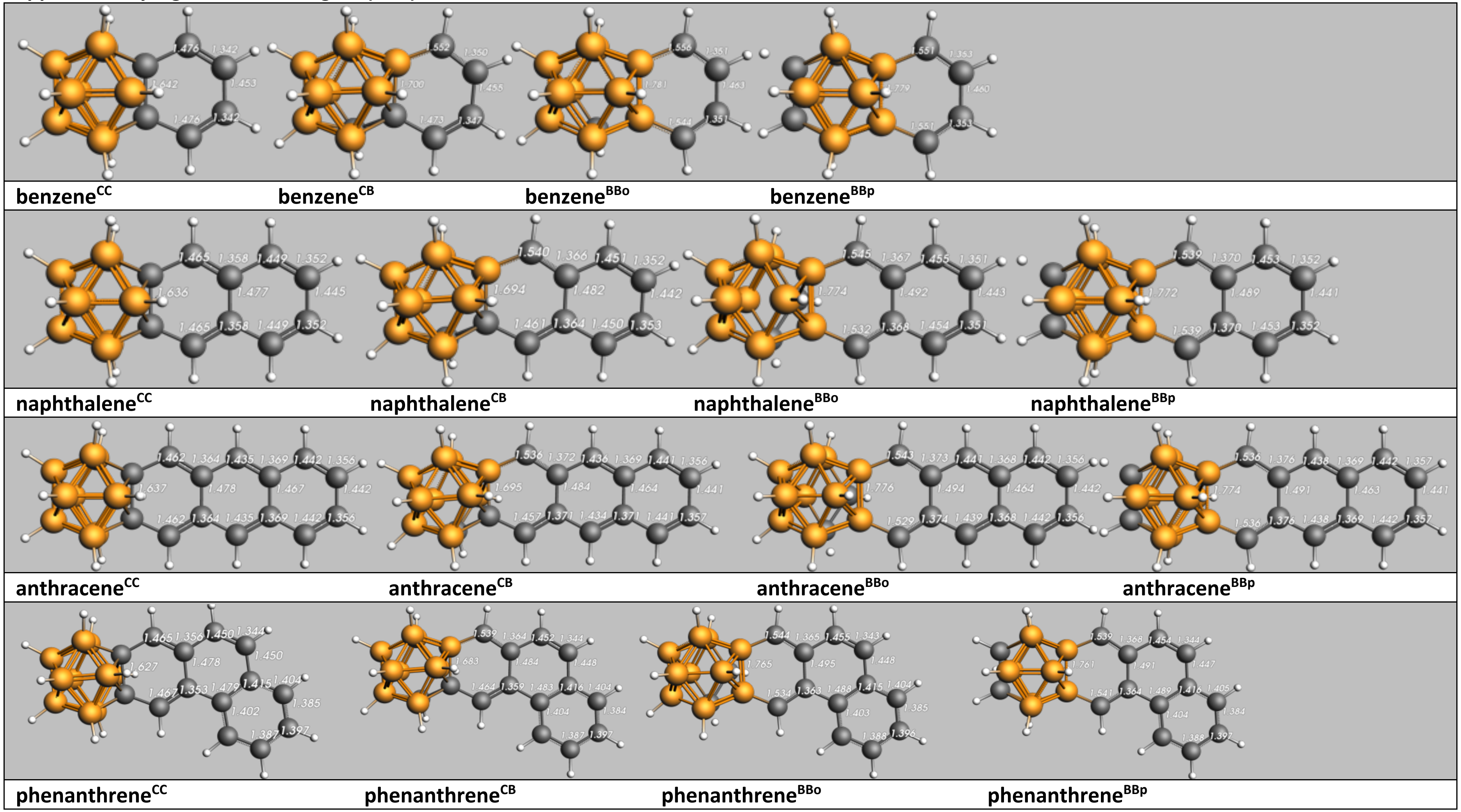

Supplementary Figure 2 QTAIM data. QTAIM localization index (left value, a.u.) and sum of delocalization indices, $\sum_{A \neq B} \delta(A, B)$, (right value, a.u.) of the 3D/3D systems formed from $[Sn_{12}]^{2-}$ and $[Sn_{10}]^{2-}$ clusters, and comparison to those for $[B_{10}H_{10}]^{2-}$.[a]

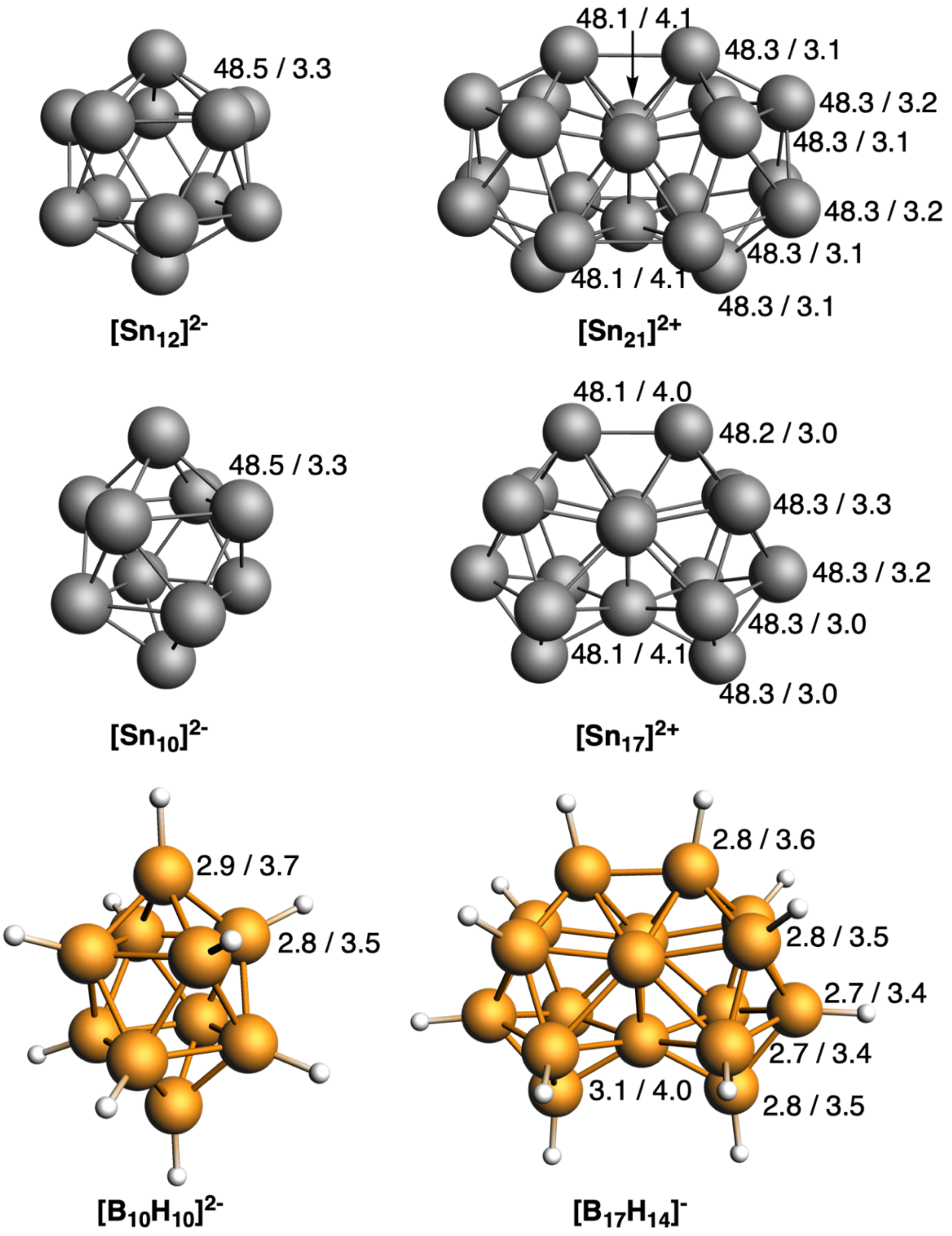

[a] To measure the electron delocalization, we have used the negative of the exchange-correlation density ($\Gamma_{XC}(\vec{r}_1, \vec{r}_2)$).[1, 2] Besides, we have employed the AIM theory[3, 4] to obtain an exhaustive physically sound subdivision of molecular space by the means of a density topological analysis. Integration of the exchange-correlation density over two atomic basins yields atomic localization indices (LIs) and delocalization indices (Dis) defined as:[1, 2]

$$\lambda(A) = -\int_A d\vec{r}_1 \int_A d\vec{r}_2 \Gamma_{XC}(\vec{r}_1, \vec{r}_2) \quad (1)$$

and

$$\delta(A,B) = -2\int_A d\vec{r}_1 \int_B d\vec{r}_2 \Gamma_{XC}(\vec{r}_1, \vec{r}_2) \ , \quad (2)$$

respectively.

The AIM topological analysis provides an exhaustive partition of molecular space, so these indices obey the following sum rule:

$$\frac{1}{2}\sum_{A \neq B} \delta(A,B) + \lambda(A) = N(A) \qquad , \quad (3)$$

where N(A) is the average population of an atom A defined as follows:

$$\langle N \rangle_A = N(A) = \int_A \rho(\vec{r}) d\vec{r} . \quad (4)$$

The DI measure, $\delta(A,B)$, provides a quantitative idea of the number of electrons delocalized or shared between atoms A and B, whereas $\sum_{A \neq B} \delta(A,B)$ provides a measure of the electrons delocalized between A and the rest of the atoms of the molecule.[1, 2]

As can be seen in Supplementary Fig. 2, the number of delocalized electrons per B or Sn atom, i.e., those electrons that contribute to the aromaticity of the cluster, is quite similar.

SUPPLEMENTARY DISCUSSION

Overlap between the π-MOs of the PAH and the n+1 MOs in the cage

In the manuscript we state: "The main reason for non-having a 3D/2D aromatic species is the lack of overlap between the π-MOs of the PAH and the n+1 MOs in the cage that deter a higher electronic delocalization". So, with the aim to further justify this lack of overlap, a model system has been built. In particular, from benzene^{CC} (Fig. 7 in the manuscript), the two C-C bonds linking the carborane and the benzene have been broken, and its interaction has been analyzed by means of a Kohn-Sham molecular orbital analysis together with an energy decomposition analysis (Supplementary Fig. 3). Both fragments have 2 unpaired electrons (triplet state) to form the two broken C-C bonds, and the analysis has been performed at the B3LYP-D3(BJ)/TZ2P level of theory with AMS software.

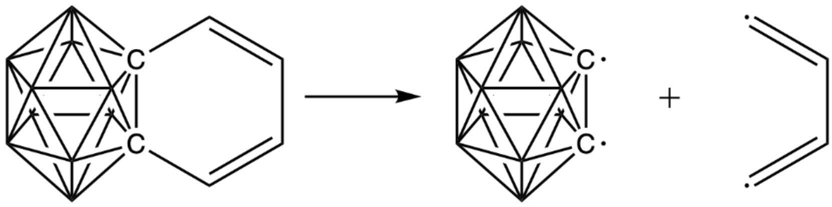

benzene^{CC} **carboryne$^{\alpha\alpha}$** ***cis*-1,3-butadienyl$^{\beta\beta}$**

Supplementary Figure 3 EDA model. Model system used in the energy decomposition analysis.

Supplementary Table 4. Overlap matrix between the fragment molecular orbitals (FMOs) of the two fragments considered in the EDA analysis. FMOs energies (in eV) also enclosed.

								**carboryne$^{\alpha\alpha}$**
		-9.31	-9.30	-9.25	-9.22	-7.23	-6.02	-0.68
***cis*-1,3-butadieny$^{\beta\beta}$**		**HOMO-4**	**HOMO-3**	**HOMO-2**	**HOMO-1**	**SOMO**	**SOMO'**	**LUMO**
-11.55	**HOMO-4**	0.028	0.001	0.000	0.000	0.000	0.052	0.000
-11.23	**HOMO-3**	0.000	0.000	0.031	0.000	0.136	0.000	0.000
-9.35	**HOMO-2**	0.000	0.000	0.000	0.051	0.000	0.000	0.000
-6.68	**HOMO-1**	0.002	0.043	0.000	0.000	0.000	0.000	0.113
-5.61	**SOMO**	0.000	0.000	0.018	0.000	0.330	0.000	0.000
-5.00	**SOMO'**	0.028	0.001	0.000	0.000	0.000	0.429	0.000
-1.52	**LUMO**	0.000	0.000	0.000	0.052	0.000	0.000	0.000

The favorable interaction between these two fragments amounts to ΔE_{int} = -258.2 kcal/mol, which can be divided into ΔE_{Pauli} = 576.5, ΔV_{elstat} = -347.3, ΔE_{oi} = -473.3 and ΔE_{disp} = -13.2 kcal/mol. This interaction mainly involves the formation of the two C-C bonds between the

two single-occupied molecular orbitals of each fragment (SOMO and SOMO’, Supplementary Fig. 4), which also implies an important overlap (<SOMO | SOMO> = 0.330 and <SOMO’ | SOMO’> = 0.429, Table 4).

However, we are interested in how the π molecular orbitals of the benzenoid interact with the MOs of the carborane, and for such we have to focus on HOMO-1 and HOMO-2 FMOs of the *cis*-1,3-butadienyl fragment (Supplementary Fig. 4). And we can see that both of them present poor overlap with the FMOs of the carborane (Table S7, the maximum overlap is 0.113 between the HOMO-1 of the *cis*-1,3-butadienyl and the LUMO of the carboryne), thus confirming our initial statement.

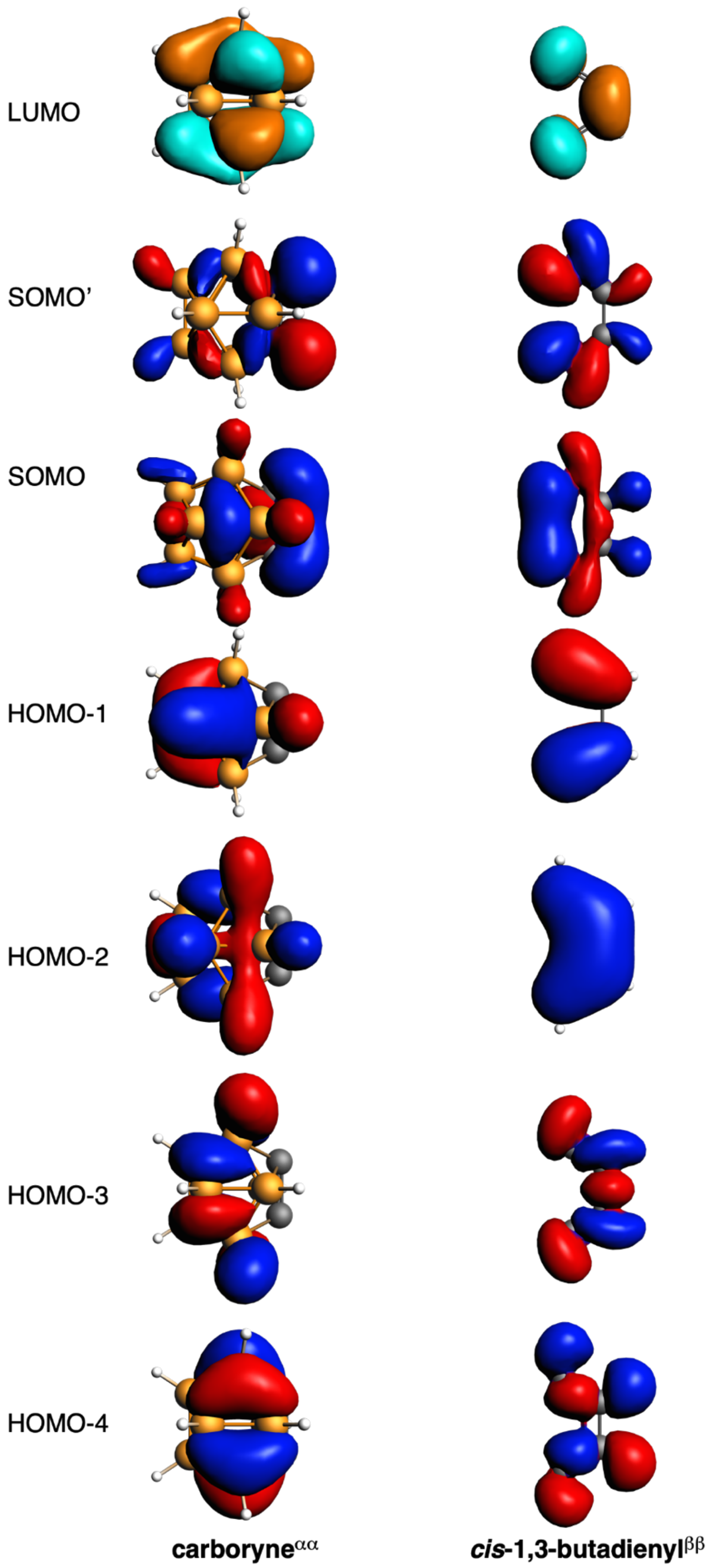

Supplementary Figure 4 FMOs of each fragment.

Supplementary References

1. Fradera X, Austen MA, Bader RFW. The Lewis model and beyond. *J Phys Chem A* **103**, 304-314 (1999).

2. Fradera X, Poater J, Simon S, Duran M, Solà M. Electron-pairing analysis from localization and delocalization indices in the framework of the atoms-in-molecules theory. *Theor Chem Acc* **108**, 214-224 (2002).

3. Bader RFW. Atoms in Molecules. *Acc Chem Res* **18**, 9-15 (1985).

4. Bader RFW. A Quantum-Theory of Molecular-Structure and its Applications. *Chem Rev* **91**, 893-928 (1991).
