# Supplementary material for: 3D and 2D aromatic units behave like oil and water in the case of benzocarborane derivatives
Source: Nat Commun. 2022 Jul 4;13:3844. doi: 10.1038/s41467-022-31267-7 (PMC9253344; doi:10.1038/s41467-022-31267-7)
Supplement: Supplementary file 2 — Description of Additional Supplementary Files [file 41467_2022_31267_MOESM2_ESM.docx]

**DESCRIPTION OF ADDITIONAL SUPPLEMENTARY FILES**

File Name: Supplementary Data 1

Description: The file includes the cartesian coordinates of all computed compounds. They are divided into:

**Cartesian coordinates of 3D/3D fused systems.**

**Cartesian coordinates of fused systems between carborane and PAHs.**

**Cartesian coordinates of reference aromatic systems.**
