# Supplementary material for: 3D and 2D aromatic units behave like oil and water in the case of benzocarborane derivatives
Source: Nat Commun. 2022 Jul 4;13:3844. doi: 10.1038/s41467-022-31267-7 (PMC9253344; doi:10.1038/s41467-022-31267-7)
Supplement: Supplementary file 3 — Supplementary Data 1 [file 41467_2022_31267_MOESM3_ESM.docx]

**SUPPLEMENTARY DATA 1**

**Cartesian coordinates of 3D/3D fused systems.**

B7H7-2

5 0.000000 0.000000 1.167681

1 0.000000 0.000000 2.381646

5 0.000000 1.408863 0.000000

5 -0.828109 -1.139794 0.000000

5 0.828109 -1.139794 0.000000

5 1.339909 0.435363 0.000000

5 0.000000 0.000000 -1.167681

1 0.000000 0.000000 -2.381646

5 -1.339909 0.435363 0.000000

1 1.540672 -2.120553 0.000000

1 -1.540672 -2.120553 0.000000

1 -2.492859 0.809979 0.000000

1 0.000000 2.621147 0.000000

1 2.492859 0.809979 0.000000

B12H10-2

1 0.000000 0.000000 3.779890

1 -2.479715 0.001425 2.002166

1 2.479715 -0.001425 -2.002166

1 0.000000 0.000000 -3.779890

1 -2.479715 0.001425 -2.002166

1 2.479715 -0.001425 2.002166

5 1.347403 -0.001018 1.603070

5 0.869894 -0.001990 0.000000

5 -0.869894 0.001990 0.000000

5 -1.347403 0.001018 1.603070

5 0.000000 0.000000 2.580677

5 1.347403 -0.001018 -1.603070

5 0.000000 0.000000 -2.580677

5 -1.347403 0.001018 -1.603070

5 0.000000 -1.157995 1.268104

5 0.000000 -1.157995 -1.268104

5 0.000000 1.157995 1.268104

5 0.000000 1.157995 -1.268104

1 -0.001855 -2.348191 1.203086

1 -0.001855 -2.348191 -1.203086

1 0.001855 2.348191 1.203086

1 0.001855 2.348191 -1.203086

B11H18-

5 -1.594585 -0.001100 0.892069

1 -2.426810 -0.001440 1.746409

5 0.000622 -0.804107 0.951831

5 -1.098177 1.382044 -0.178564

5 -1.743599 0.000034 -0.900488

5 -1.096585 -1.382160 -0.179032

5 -0.000287 -0.000823 -0.835436

1 -1.376321 -2.503534 -0.445227

1 1.379369 -2.503345 -0.443103

1 -2.627798 -0.002270 -1.696274

1 -1.378729 2.503795 -0.442323

5 1.594893 0.001146 0.891774

1 2.426743 0.001522 1.746431

5 -0.000418 0.803571 0.951227

5 1.098567 -1.381874 -0.178457

5 1.743233 0.000723 -0.900772

5 1.096470 1.382552 -0.179138

1 2.627153 0.000909 -1.696899

1 1.375725 2.504341 -0.444078

B12H12-2

5 0.891722 -1.227318 0.758691

1 1.522762 -2.095650 1.295212

5 -0.891734 -1.227361 0.758726

1 -1.522803 -2.095725 1.295154

5 0.000013 -1.516450 -0.758301

1 -0.000086 -2.589799 -1.295106

5 0.000032 0.000108 -1.696133

1 -0.000107 -0.000189 -2.896190

5 -0.000032 -0.000108 1.696133

1 0.000107 0.000189 2.896190

5 -0.000013 1.516450 0.758301

1 0.000086 2.589799 1.295106

5 -1.442896 0.468687 0.758576

1 -2.463737 0.800520 1.295162

5 -1.442671 -0.468557 -0.758431

1 -2.463457 -0.800523 -1.295126

5 1.442671 0.468557 0.758431

1 2.463457 0.800523 1.295126

5 1.442896 -0.468687 -0.758576

1 2.463737 -0.800520 -1.295162

5 -0.891722 1.227318 -0.758691

1 -1.522762 2.095650 -1.295212

5 0.891734 1.227361 -0.758726

1 1.522803 2.095725 -1.295154

B21H18-

1 -1.041886 -0.232089 -2.876382

5 2.738360 -0.830940 0.574858

5 -2.738245 -0.831444 0.574362

1 3.653409 -1.450725 1.003317

5 -1.154077 -0.138667 -1.713586

1 3.652607 -0.142212 -1.758920

5 1.682387 -1.514112 -0.717133

1 1.886234 -2.568984 -1.217412

5 1.153639 1.553751 0.736065

1 1.041740 2.607887 1.235794

5 2.737524 0.914087 0.432538

1 3.651983 1.595595 0.755454

5 1.681512 1.378059 -0.952512

1 1.884384 2.338838 -1.616109

5 1.154496 -0.139475 -1.713652

1 1.042254 -0.234071 -2.876346

5 1.681961 0.135925 1.669708

1 1.885255 0.230783 2.833444

5 1.155045 -1.414899 0.977620

1 1.042426 -2.374537 1.640874

5 2.738002 -0.081642 -1.007380

5 -0.000098 0.856131 -0.592103

5 -2.737819 -0.081622 -1.007559

5 -0.000095 0.083799 1.037864

1 -3.652359 -0.142246 -1.759174

5 0.000000 -0.941795 -0.445604

1 -3.653226 -1.451442 1.002652

5 -1.682229 -1.513991 -0.717755

1 -1.886019 -2.568686 -1.218427

5 -1.154234 1.553773 0.736927

1 -1.042634 2.607696 1.237164

5 -2.737674 0.913613 0.432687

1 -3.652425 1.594795 0.755506

5 -1.682078 0.135332 1.669712

1 -1.885512 0.229545 2.833474

5 -1.154675 -1.415177 0.976974

1 -1.042152 -2.374909 1.640117

5 -1.681604 1.378396 -0.951953

1 -1.884567 2.339249 -1.615415

B10H14

5 -0.000030 0.890795 1.090425

5 0.000064 -0.890467 1.090668

5 -1.496368 -0.000086 0.715642

5 0.990292 1.419947 -0.247321

5 -1.786890 -0.000199 -0.981587

5 -0.990502 1.419920 -0.247217

5 1.786843 -0.000024 -0.981602

5 -0.990051 -1.420003 -0.247108

5 1.496382 0.000103 0.715621

5 0.990240 -1.419949 -0.247117

1 -1.017652 0.965658 -1.482516

1 -1.017852 -0.966114 -1.482535

1 1.017770 0.965793 -1.482667

1 1.018025 -0.966071 -1.482564

1 -2.796164 -0.000043 -1.591930

1 2.796093 0.000037 -1.592002

1 -1.525923 2.470159 -0.295959

1 1.525724 2.470188 -0.296047

1 1.525730 -2.470178 -0.295521

1 0.000084 -1.620738 2.020010

1 -0.000065 1.621067 2.019769

1 2.397010 0.000153 1.477632

1 -2.396955 0.000041 1.477703

1 -1.525724 -2.470146 -0.295396

B20H16

5 2.645630 0.000822 -0.877041

1 3.667036 0.001874 -1.467756

5 2.645628 -0.000821 0.876985

1 3.667012 -0.001837 1.467740

5 1.994221 -1.419658 -0.001431

1 2.483482 -2.493504 -0.003087

5 0.257447 -1.192511 -0.001403

5 -1.249035 -1.548085 0.877498

5 1.994186 1.419656 0.001456

1 2.483414 2.493517 0.003315

5 0.257439 1.192459 0.001355

1 -1.221919 -2.451702 1.630708

5 1.249022 0.877469 1.548016

1 1.222038 1.630488 2.451792

5 1.248562 -0.880927 1.545793

1 1.222024 -1.637199 2.446966

5 1.248521 0.880942 -1.545886

1 1.221898 1.637276 -2.446999

5 1.249037 -0.877461 -1.548081

1 1.221954 -1.630564 -2.451786

5 -0.257503 -0.001439 1.192420

1 -1.221966 -2.446970 -1.637363

5 -0.257435 0.001337 -1.192215

5 -1.248625 -1.545903 -0.880979

1 -1.221998 2.451685 -1.630761

5 -1.249015 1.548093 -0.877526

1 -1.221850 2.446935 1.637376

5 -1.248428 1.545886 0.880959

1 -3.666979 1.467896 0.001847

5 -2.645608 0.877124 0.000805

5 -1.994200 0.001433 -1.419516

1 -2.483507 0.003198 -2.493347

1 -3.667088 -1.467756 -0.001776

5 -2.645680 -0.877051 -0.000812

5 -1.994177 -0.001415 1.419550

1 -2.483476 -0.003091 2.493389

B10H10-2

5 0.000000 1.301050 0.760748

5 1.301050 0.000000 0.760748

5 0.000000 0.000000 1.856523

5 -1.301050 0.000000 0.760748

5 -0.919981 0.919981 -0.760748

5 -0.919981 -0.919981 -0.760748

5 0.000000 0.000000 -1.856523

5 0.919981 -0.919981 -0.760748

5 0.919981 0.919981 -0.760748

5 0.000000 -1.301050 0.760748

1 0.000000 0.000000 3.056349

1 0.000000 -2.434849 1.164802

1 2.434849 0.000000 1.164802

1 0.000000 2.434849 1.164802

1 1.721699 1.721699 -1.164802

1 1.721699 -1.721699 -1.164802

1 0.000000 0.000000 -3.056349

1 -1.721699 -1.721699 -1.164802

1 -1.721699 1.721699 -1.164802

1 -2.434849 0.000000 1.164802

B17H14-1

5 1.845834 0.869265 0.968655

1 -1.609461 -2.982179 -0.000523

5 0.888000 1.903163 -0.000399

5 1.845501 0.868970 -0.969153

5 2.568674 -0.479062 -0.000234

5 1.391975 -0.769373 -1.325212

5 1.495444 -1.803953 0.000292

1 -3.753574 -0.563963 -0.000272

5 1.392740 -0.768974 1.325479

5 -0.000185 0.614006 -0.909613

1 1.118574 3.058180 -0.000675

1 3.753647 -0.563659 -0.000517

1 1.399519 -1.152536 -2.444663

1 2.529617 1.504821 1.702663

1 1.400735 -1.151622 2.445113

1 2.529041 1.504161 -1.703704

1 1.609651 -2.982096 0.000377

5 0.000053 0.614180 0.909892

5 -1.845812 0.868896 0.969000

5 -0.888145 1.903191 0.000562

1 -1.400167 -1.152479 2.444706

5 0.000017 -0.954569 0.000512

5 -1.392102 -0.768921 -1.325506

5 -1.495328 -1.804032 -0.000297

5 -2.568611 -0.479197 -0.000109

5 -1.392381 -0.769500 1.325187

5 -1.845657 0.869172 -0.968743

1 -1.118826 3.058186 0.000780

1 -2.529188 1.504642 -1.703057

1 -2.529748 1.503974 1.703286

1 -1.399904 -1.151743 -2.445074

CB11H12-1

5 0.663692 1.353046 0.743285

1 1.096163 2.237583 1.403657

5 -0.248022 1.499146 -0.767674

1 -0.424191 2.560082 -1.273051

5 -1.088459 1.054554 0.726131

1 -1.802491 1.743126 1.375366

5 -1.345339 -0.703705 0.719831

1 -2.227071 -1.164977 1.364327

5 1.352767 0.706899 -0.754792

1 2.309305 1.207715 -1.251080

5 1.093559 -1.060012 -0.761324

1 1.867214 -1.810353 -1.261790

5 0.015855 0.003823 -1.701413

1 0.026748 0.006675 -2.890451

5 -1.495677 0.221997 -0.782619

1 -2.554556 0.378601 -1.298739

5 -0.666743 -1.359283 -0.777837

1 -1.138161 -2.321835 -1.291090

1 0.406865 -2.467441 1.386704

6 -0.014036 -0.003321 1.518524

5 1.489066 -0.220632 0.746753

5 0.246698 -1.491300 0.732975

1 2.461280 -0.365779 1.409203

1 -0.023871 -0.006137 2.599228

C2B10H12

5 -1.4551070 0.0022680 0.8507330

1 -2.3295020 0.0040570 1.6367450

5 -1.4495070 0.0009390 -0.9105340

1 -2.4714050 0.0018550 -1.5002560

5 -0.8923120 -1.4360610 -0.0258880

1 -1.4781580 -2.4444450 0.1396570

5 0.8889490 -1.4382130 -0.0238410

1 1.4729040 -2.4474700 0.1429990

5 -0.8888790 1.4382160 -0.0280700

1 -1.4725280 2.4481840 0.1354980

5 0.8923370 1.4360840 -0.0264640

1 1.4785180 2.4443160 0.1387970

5 0.0027240 0.8884960 -1.4601010

1 0.0047150 1.5290790 -2.4514080

5 0.0005780 -0.8909430 -1.4586970

1 0.0010400 -1.5334220 -2.4487750

5 1.4512580 -0.0026580 -0.9071950

1 2.4744190 -0.0036870 -1.4947590

1 2.3257740 -0.0016110 1.6416870

6 -0.0023720 -0.8101780 1.2724250

6 -0.0004300 0.8123300 1.2711600

5 1.4531100 -0.0008230 0.8537380

1 -0.0009430 1.2894660 2.2389760

1 -0.0037730 -1.2857530 2.2409240

CB20H18

1 -0.998709 2.505602 1.463022

5 2.744989 -0.000955 -0.991404

5 -2.744992 0.000853 -0.991418

1 3.675242 -0.001397 -1.716637

5 -1.170881 1.550690 0.818783

1 3.598612 1.502104 0.970562

5 1.690189 1.451228 -0.850916

1 1.871129 2.455819 -1.443289

5 1.170892 -1.550547 0.818921

1 0.998933 -2.505318 1.463372

5 2.702955 -0.872657 0.529770

1 3.597818 -1.502471 0.971924

5 1.549147 0.000867 1.648608

1 1.632761 0.001311 2.823569

5 1.172069 1.551969 0.817152

1 1.000491 2.507887 1.460065

5 1.689619 -1.452451 -0.849443

1 1.870258 -2.457835 -1.440556

5 1.162320 -0.001018 -1.722792

1 1.040357 -0.001515 -2.884232

5 2.703447 0.872406 0.528833

6 0.000039 0.000023 0.939596

5 -2.702914 0.872748 0.529689

5 -0.000184 -0.922345 -0.533422

1 -3.597728 1.502636 0.971843

5 0.000179 0.922256 -0.533472

1 -3.675243 0.001237 -1.716652

5 -1.689592 1.452361 -0.849587

1 -1.870187 2.457679 -1.440824

5 -1.172215 -1.551985 0.817323

1 -1.000793 -2.507877 1.460254

5 -2.703487 -0.872291 0.528924

1 -3.598677 -1.501904 0.970727

5 -1.690225 -1.451331 -0.850760

1 -1.871204 -2.455965 -1.443050

5 -1.162305 0.000833 -1.722767

1 -1.040296 0.001292 -2.884201

5 -1.549072 -0.000699 1.648576

1 -1.632695 -0.001083 2.823540

C2B19H18+1

1 0.766568 0.966024 -2.669996

5 -2.641368 0.354117 1.022249

5 2.739553 0.609229 0.723324

1 -3.530614 0.622325 1.743468

5 1.032291 0.644898 -1.580713

1 -3.684132 0.970741 -1.331093

5 -1.669718 1.680295 0.270655

1 -1.812391 2.826924 0.503221

5 -1.210701 -1.789844 -0.317146

1 -0.903213 -2.892856 -0.528045

5 -2.681513 -1.019721 -0.085035

1 -3.590439 -1.768076 -0.119970

5 -1.613848 -0.583375 -1.511027

1 -1.653047 -1.007275 -2.607523

5 -1.302497 1.252021 -1.356004

1 -1.104005 1.911380 -2.296877

5 -1.537729 -1.044986 1.326562

1 -1.566498 -1.742675 2.270923

5 -1.027092 0.646211 1.577799

1 -0.758501 0.965662 2.666943

5 -2.738896 0.612585 -0.723526

6 -0.011612 -0.525547 -0.787760

5 2.642484 0.347743 -1.021607

6 0.005502 -0.530819 0.781486

1 3.533422 0.613856 -1.741574

5 0.001224 1.114828 0.000334

1 3.685563 0.964007 1.331993

5 1.673403 1.678576 -0.272493

1 1.817731 2.824507 -0.507872

5 1.206754 -1.790613 0.324029

1 0.902283 -2.894449 0.535492

5 2.677752 -1.024798 0.086982

1 3.586291 -1.773604 0.120853

5 1.612465 -0.581134 1.512051

1 1.644036 -1.003962 2.609256

5 1.309012 1.258419 1.355692

1 1.111084 1.921264 2.294219

5 1.534159 -1.048097 -1.325540

1 1.563842 -1.747359 -2.268688

Sn12-2

Sn -2.62137123 0.85173515 -1.37925669

Sn 1.62009652 2.22987156 1.37925669

Sn 1.62009652 -2.22987156 -1.37925669

Sn 0.00000000 -2.75627282 1.37925669

Sn 0.00000000 0.00000000 3.08554087

Sn -2.62137123 -0.85173515 1.37925669

Sn 2.62137123 -0.85173515 1.37925669

Sn -1.62009652 2.22987156 1.37925669

Sn 0.00000000 2.75627282 -1.37925669

Sn 2.62137123 0.85173515 -1.37925669

Sn 0.00000000 0.00000000 -3.08554087

Sn -1.62009652 -2.22987156 -1.37925669

Sn10-2

Sn 4.08666929 0.57465542 -1.91352206

Sn 3.16330788 -1.65495387 -3.87654525

Sn 0.93369517 -0.73160089 -1.91352291

Sn 2.23994954 -3.88456566 -1.91352623

Sn 5.39291816 -2.57831622 -1.91352282

Sn 5.39292013 -0.73159758 0.59111060

Sn 2.23994975 0.57465424 0.59110798

Sn 0.93370101 -2.57831728 0.59111198

Sn 3.16330977 -1.65495381 2.55413958

Sn 4.08666304 -3.88456567 0.59111173

Sn21+2

Sn -2.3511912 0.7700128 -1.4438189

Sn 1.8083894 2.3981837 1.4568262

Sn 1.6205236 -2.2009800 -1.3254336

Sn 0.0209288 -2.9832996 1.5084416

Sn 0.2768562 -0.0633523 3.0864246

Sn -2.3662789 -0.9095179 1.3301236

Sn 5.3940176 0.9768713 -2.5173809

Sn -1.3424652 2.1695093 1.2991545

Sn 0.3067045 2.7071471 -1.4958907

Sn 7.2469377 -0.7208943 -0.3961756

Sn 0.0406536 -0.0472538 -3.3412985

Sn -1.3863363 -2.3753071 -1.4429578

Sn 2.6148684 0.7736138 -1.3721665

Sn 5.3617316 -1.7492814 2.0939559

Sn 6.2325071 -2.1417977 -3.1289946

Sn 3.6913063 -4.0991823 -2.4171583

Sn 3.3066460 -4.0742736 0.8904940

Sn 3.3410363 -1.1888795 -3.9910845

Sn 6.2139100 -3.7928189 -0.3354926

Sn 2.5966173 -0.8312383 1.3198135

Sn 5.1163075 1.2963488 0.7865076

Sn17+2

Sn 3.81456847 0.64471113 -1.97067452

Sn 3.29652032 -1.99208396 -4.16644609

Sn 1.01656941 -0.67209308 -1.99481957

Sn 2.05904136 -3.66817202 -1.91649358

Sn 5.35248512 -2.17055902 -1.89827325

Sn 5.38662066 -0.46203591 0.68706277

Sn 2.15837500 0.50271221 0.54736412

Sn 0.74768424 -2.56687230 0.66905270

Sn 3.09131250 -1.56802131 2.56902931

Sn 4.03985084 -3.64052228 0.54133542

Sn 2.77137698 3.40057324 -3.27044488

Sn 2.03141623 0.92737538 -4.90176707

Sn -0.47197264 1.79096806 -3.32815000

Sn 0.87853729 3.13122016 1.38278624

Sn -1.03503205 1.24404495 -0.33334653

Sn 3.55074944 3.45736553 -0.27301316

Sn 0.36484578 4.19165714 -1.43502325

B13H10-1

5 1.0076700 1.1525010 0.9353050

5 -0.5765740 0.3411550 0.9447000

5 -0.3326170 1.7634120 -0.0000710

5 1.0074010 1.1520970 -0.9357240

5 2.0325400 -0.0390100 -0.0003600

5 0.9310500 -0.6042520 -1.3191700

5 1.3058370 -1.6168800 0.0001230

5 -0.2992090 -1.1070270 0.0009730

5 0.9320550 -0.6035600 1.3191970

5 -0.5766870 0.3411200 -0.9445860

1 -0.7317960 2.8757230 -0.0002560

5 -1.8680500 -0.8267840 0.8415420

5 -2.0128790 0.7094840 0.0000990

1 1.5161910 1.9219100 1.6859360

1 1.0614180 -0.9788780 2.4381180

1 -2.8779760 1.5178440 -0.0001780

1 1.7999600 -2.6949070 0.0003190

1 1.0601880 -0.9799560 -2.4379820

1 3.2127180 0.1099690 -0.0006050

1 1.5154200 1.9211100 -1.6871020

5 -1.8675020 -0.8269290 -0.8416490

1 -2.4850400 -1.4351300 -1.6513870

1 -2.4862550 -1.4343230 1.6512370

B15H12-1

5 -0.777456 -0.64974 0.761916

1 -0.0464970 1.8614530 -2.1816010

5 0.8139870 -1.1060040 1.2959300

1 1.0486680 -1.8612960 2.1813050

5 0.2135370 0.5723240 1.6136840

1 -0.0461270 0.9584280 2.7028800

5 0.8144530 1.6754040 0.3097100

1 1.0488270 2.8198420 0.5213520

5 0.2126270 -1.6837600 -0.3112250

1 -0.0471910 -2.8200700 -0.5213800

5 0.8138890 -0.5694790 -1.6056480

1 1.0484270 -0.9585230 -2.7025000

5 1.8367540 -1.0073350 -0.1862790

1 2.7795740 -1.7173160 -0.3176420

5 1.8372350 0.3422440 0.9650080

1 2.7801940 0.5831590 1.6454520

5 -0.7777320 -0.3345190 -0.9435990

5 0.2131110 1.1114040 -1.3024640

5 -0.7775370 0.9848850 0.1823090

1 2.7796910 1.1329830 -1.3286720

5 1.8368180 0.6646040 -0.7792680

5 -2.2694500 0.6358670 -0.7438020

5 -2.2694940 -0.9620580 -0.1785690

5 -2.2695120 0.3263510 0.9224690

1 -2.8671030 1.2981540 -1.5194410

1 -2.8669470 -1.9650830 -0.3640270

1 -2.8676560 0.6673420 1.8834160

B6H6-2

5 -0.3419630 -1.1753710 0.0975980

5 -0.8837040 0.3222800 0.7897550

5 -0.7811020 0.1491260 -0.9359250

5 0.3410560 1.1757720 -0.0976940

5 0.8840410 -0.3223490 -0.7891900

5 0.7814980 -0.1493020 0.9354300

1 -0.6790720 -2.3357620 0.1939720

1 1.5531040 -0.2972070 1.8585590

1 1.7566880 -0.6409920 -1.5678140

1 0.6798390 2.3356680 -0.1939190

1 -1.5530390 0.2968360 -1.8587950

1 -1.7566500 0.6406760 1.5681280

**Cartesian coordinates of fused systems between carborane and PAHs.**

benzeneCC

5 -1.786004 0.000382 -1.443415

1 -2.365622 0.000783 -2.471472

5 -2.329025 0.893687 0.000026

1 -3.318683 1.536940 0.000430

5 -0.895235 1.434112 -0.889746

1 -0.726380 2.444462 -1.473134

6 0.427239 0.820863 0.000481

1 3.845364 1.236278 -0.000195

5 -2.329138 -0.893611 -0.000381

1 -3.318839 -1.536796 -0.000434

5 -0.895636 -1.434174 0.889613

1 -0.727110 -2.444169 1.473701

5 -1.786304 -0.000296 1.443119

1 -2.366263 -0.000356 2.470975

5 -0.895390 1.433733 0.890195

1 -0.727263 2.443498 1.474790

5 -0.895358 -1.433741 -0.890375

1 -0.726404 -2.443708 -1.474400

5 -0.034081 0.000270 -1.461204

1 0.750325 0.000459 -2.335096

5 -0.034226 -0.000414 1.461095

1 0.749218 -0.000560 2.335861

6 0.427188 -0.820918 0.000025

1 1.776346 2.535582 -0.000055

1 3.845296 -1.236327 0.000213

6 1.761238 -1.453585 -0.000008

6 2.889478 -0.726631 -0.000047

6 2.889513 0.726656 -0.000084

6 1.761243 1.453575 0.000281

1 1.776601 -2.535586 0.000294

benzeneCB

5 -1.829472 -0.010597 1.432501

1 -2.452445 -0.072900 2.432755

5 -2.374283 0.879978 -0.014006

1 -3.406848 1.451007 -0.041733

5 -2.316317 -0.902841 -0.022360

1 -3.207139 -1.667340 -0.119969

6 -0.864353 -1.263934 -0.810008

1 -0.729198 -2.187522 -1.352553

5 -0.965378 1.463463 0.904746

1 -0.978831 2.462281 1.534882

5 0.506659 0.958248 0.037298

1 3.810621 -1.361399 -0.020623

5 -0.932432 1.476566 -0.883788

1 -0.919198 2.468904 -1.523326

5 -1.781577 0.021965 -1.443063

1 -2.303136 -0.139256 -2.487025

5 -0.063912 0.050089 1.468689

1 0.646980 -0.041781 2.402333

5 -0.889505 -1.406471 0.901837

1 -0.746335 -2.477678 1.366185

5 -0.016920 0.080912 -1.425174

1 0.678912 -0.080658 -2.357634

6 0.443196 -0.740887 0.037286

1 1.703714 -2.544357 -0.011968

1 4.028441 1.058682 -0.005699

6 1.959296 1.503766 0.023609

6 3.016961 0.664660 0.003609

6 2.891680 -0.785018 -0.011960

6 1.727298 -1.461546 -0.012707

1 2.145689 2.573215 0.021998

benzeneBBo

5 -1.726895 0.021604 -1.484944

1 2.078398 2.589900 0.102010

1 4.007435 1.193501 -0.079684

1 3.997706 -1.205179 -0.107608

6 1.919867 -1.513564 0.036308

1 -2.096872 -0.101897 -2.594180

6 1.930588 1.514428 0.067174

6 3.025808 0.729883 -0.029750

6 3.020257 -0.733500 -0.046978

1 2.051722 -2.591283 0.058553

5 -0.166402 0.010969 1.529802

1 0.408357 -0.005591 2.559743

5 -1.937170 -0.005730 1.374717

1 -2.642917 -0.041037 2.320203

5 -0.986252 -1.430964 0.900309

1 -0.990703 -2.512212 1.369319

6 -0.855631 -1.291336 -0.783276

1 -0.733125 -2.153461 -1.421316

5 -1.021019 1.449416 0.905636

1 -1.067481 2.460204 1.514730

5 -0.883241 1.455923 -0.862405

1 -0.746123 2.371878 -1.591207

5 -2.386163 0.887760 -0.106552

1 -3.400609 1.479704 -0.219290

5 -2.354115 -0.896655 -0.103122

1 -3.262568 -1.622036 -0.292534

5 0.495265 0.919068 0.140443

1 0.591683 -0.053214 -2.079623

5 0.525699 -0.861436 0.156894

6 -0.039795 0.034245 -1.208980

benzeneBBp

5 0.042496 -0.000124 1.453195

1 -0.565955 -0.000267 2.463181

5 -0.531529 -0.889631 0.000315

1 -4.032505 -1.196067 -0.000234

5 0.908259 -1.441486 0.892672

1 1.073764 -2.449427 1.480448

6 2.204564 -0.818047 -0.000368

1 3.174640 -1.289600 -0.000583

5 -0.531430 0.889402 0.000427

1 -2.098409 -2.594342 0.000455

5 0.907805 1.441467 -0.892724

1 1.072799 2.449379 -1.480687

5 0.041874 0.000099 -1.452768

1 -0.567168 0.000094 -2.462394

5 0.907861 -1.441320 -0.892962

1 1.073032 -2.449203 -1.480928

5 0.908196 1.441352 0.892892

1 1.073535 2.449135 1.480980

5 1.788214 -0.000090 1.449353

1 2.573329 -0.000272 2.324704

5 1.787605 0.000137 -1.449752

1 2.572288 0.000077 -2.325490

6 2.204547 0.818116 -0.000236

1 3.174613 1.289798 -0.000369

1 -4.032400 1.196264 -0.000328

6 -1.949564 1.517683 0.000257

6 -3.049897 0.730190 -0.000119

6 -3.049960 -0.730077 -0.000118

6 -1.949712 -1.517691 0.000188

1 -2.098184 2.594346 0.000374

naphthaleneCC

5 2.804659 -0.000056 1.442127

1 3.383031 -0.000105 2.471019

5 3.344098 0.895711 -0.000181

1 4.332949 1.540312 -0.000297

5 1.910796 1.432610 0.891393

1 1.743507 2.444439 1.472844

1 -5.284984 1.247259 -0.000274

1 -0.752038 2.530294 -0.000059

5 3.344084 -0.895746 -0.000197

1 4.332909 -1.540384 -0.000344

5 1.910507 -1.432581 -0.891452

1 1.743017 -2.444490 -1.472712

5 2.804418 -0.000025 -1.442271

1 3.382585 -0.000051 -2.471286

5 1.910594 1.432592 -0.891430

1 1.743166 2.444518 -1.472678

5 1.910717 -1.432653 0.891352

1 1.743371 -2.444506 1.472742

5 1.053315 0.000019 1.466705

1 0.268122 0.000096 2.340424

5 1.053130 0.000054 -1.466699

1 0.267154 0.000182 -2.339713

6 -0.737867 1.447696 0.000058

6 0.585156 0.818035 0.000216

6 0.585199 -0.817962 0.000196

6 -0.737844 -1.447659 0.000048

6 -4.337259 -0.722419 -0.000084

6 -4.337269 0.722384 -0.000080

6 -3.176676 1.415276 0.000076

6 -1.895559 0.738304 0.000063

6 -1.895544 -0.738297 0.000062

6 -3.176652 -1.415293 0.000073

1 -3.175754 -2.499146 0.000086

1 -3.175800 2.499129 0.000093

1 -0.751967 -2.530256 -0.000060

1 -5.284968 -1.247304 -0.000283

naphthaleneCB

1 5 0 2.865904 -0.047137 -1.427341

2 1 0 3.488719 -0.126565 -2.426552

3 5 0 3.432484 0.823169 0.022639

4 1 0 4.482321 1.361556 0.053941

5 5 0 3.316302 -0.960650 0.026779

6 1 0 4.182806 -1.752632 0.124724

7 6 0 1.854181 -1.276009 0.809659

8 1 0 1.688498 -2.196784 1.348481

9 5 0 2.044989 1.449965 -0.900278

10 1 0 2.089139 2.450110 -1.527076

11 5 0 0.555575 0.982820 -0.038507

12 1 0 -0.744472 -2.468663 -0.006564

13 5 0 2.006505 1.459508 0.889239

14 1 0 2.017825 2.451460 1.529567

15 5 0 2.809942 -0.019321 1.447200

16 1 0 3.321962 -0.198059 2.493094

17 5 0 1.103997 0.065813 -1.473787

18 1 0 0.394738 -0.000353 -2.411097

19 5 0 1.879497 -1.415705 -0.904846

20 1 0 1.706792 -2.481307 -1.372058

21 5 0 1.048300 0.089958 1.425640

22 1 0 0.343331 -0.051329 2.354793

23 6 0 0.559859 -0.711282 -0.045665

24 1 0 -5.275615 -1.375192 0.013406

25 1 0 -3.110669 -2.512482 0.010852

26 6 0 -3.162000 -1.429529 0.007768

27 6 0 -1.986884 0.785237 -0.008980

28 6 0 -1.912436 -0.694460 0.001439

29 6 0 -3.307510 1.386029 -0.002911

30 1 0 -1.015097 2.645424 -0.016919

31 6 0 -0.868398 1.568982 -0.024105

32 1 0 -3.358861 2.468987 -0.008130

33 1 0 -5.408964 1.118721 0.004165

34 6 0 -0.735666 -1.385131 0.000808

35 6 0 -4.358244 -0.798139 0.008677

36 6 0 -4.436222 0.641804 0.003591

naphthaleneBBo

1 5 0 -2.752983 0.013457 -1.508437

2 6 0 -1.069745 0.024501 -1.203242

3 5 0 -0.523215 -0.858081 0.180973

4 1 0 -0.423371 -0.068228 -2.062400

5 1 0 1.009336 2.580971 0.138851

6 1 0 -3.101266 -0.116479 -2.623950

7 1 0 5.406565 -1.252015 -0.095710

8 6 0 2.007267 0.745549 0.039654

9 6 0 2.005191 -0.746079 0.030793

10 5 0 -0.556810 0.915493 0.149852

11 5 0 -1.245353 0.020526 1.535745

12 1 0 -0.688882 0.011396 2.575755

13 5 0 -3.010870 0.002658 1.346155

14 1 0 -3.732422 -0.026791 2.279908

15 5 0 -2.051308 -1.425613 0.898424

16 1 0 -2.065371 -2.503802 1.374287

17 6 0 -1.890919 -1.295757 -0.784319

18 1 0 -1.756569 -2.161858 -1.414451

19 5 0 -2.086759 1.455017 0.884903

20 1 0 -2.143267 2.469986 1.486287

21 5 0 -1.917331 1.449970 -0.880612

22 1 0 -1.765653 2.361174 -1.612509

23 5 0 -3.433528 0.887867 -0.147789

24 1 0 -4.446091 1.478794 -0.281111

25 5 0 -3.400421 -0.899121 -0.133017

26 1 0 -4.305466 -1.626002 -0.332812

27 6 0 0.871671 1.503361 0.103930

28 1 0 3.302936 2.490876 0.010917

29 1 0 5.411785 1.248348 -0.076797

30 6 0 0.865475 -1.500165 0.093271

31 6 0 4.461821 -0.721943 -0.059931

32 6 0 4.465409 0.721226 -0.049287

33 6 0 3.302309 1.406622 -0.000927

34 1 0 0.989530 -2.579209 0.128522

35 1 0 3.299853 -2.492605 -0.022946

36 6 0 3.298393 -1.408217 -0.020413

naphthaleneBBp

1 5 0 -1.097913 -0.000546 -1.454842

2 1 0 -0.488020 -0.001004 -2.463980

3 5 0 -0.522480 -0.885945 0.000564

4 1 0 5.431984 1.250963 0.000203

5 5 0 -1.962823 -1.442071 -0.893062

6 1 0 -2.126656 -2.450919 -1.479689

7 6 0 -3.258577 -0.819914 0.000205

8 1 0 -4.228890 -1.290898 0.000273

9 5 0 -0.522569 0.886178 -0.000216

10 6 0 2.025496 0.744260 0.000037

11 5 0 -1.963148 1.442093 0.893051

12 1 0 -2.127319 2.450856 1.479730

13 5 0 -1.098203 0.000594 1.455040

14 1 0 -0.488716 0.001235 2.464425

15 5 0 -1.962995 -1.441395 0.894255

16 1 0 -2.127104 -2.449760 1.481634

17 5 0 -1.962960 1.441363 -0.894295

18 1 0 -2.126898 2.449760 -1.481671

19 5 0 -2.841452 -0.000602 -1.449441

20 1 0 -3.624733 -0.001112 -2.326395

21 5 0 -2.841743 0.000521 1.449225

22 1 0 -3.625237 0.000898 2.325988

23 6 0 -3.258632 0.819807 -0.000524

24 1 0 -4.228978 1.290728 -0.000767

25 1 0 1.026824 -2.583825 0.000137

26 6 0 0.886397 1.505404 -0.000245

27 6 0 3.319857 1.404562 0.000181

28 6 0 4.486566 0.720677 0.000091

29 6 0 4.486539 -0.720731 -0.000177

30 6 0 3.319796 -1.404579 -0.000188

31 1 0 3.320439 2.489230 0.000340

32 1 0 1.026992 2.583883 -0.000322

33 1 0 3.320387 -2.489251 -0.000381

34 1 0 5.431932 -1.251061 -0.000393

35 6 0 2.025483 -0.744275 0.000098

36 6 0 0.886313 -1.505323 0.000433

anthraceneCC

5 -3.892934 0.000016 1.441654

1 -4.470378 0.000027 2.471143

5 -4.431254 -0.896448 -0.000038

1 -5.420171 -1.541059 -0.000057

5 -2.998231 -1.432282 0.891993

1 -2.830597 -2.444195 1.473361

6 4.539396 -1.414515 0.000000

1 -0.337937 -2.530861 0.000030

5 -4.431253 0.896452 -0.000053

1 -5.420165 1.541070 -0.000087

5 -2.998166 1.432278 -0.892020

1 -2.830486 2.444218 -1.473328

5 -3.892858 -0.000010 -1.441712

1 -4.470239 -0.000018 -2.471240

5 -2.998174 -1.432292 -0.891995

1 -2.830497 -2.444244 -1.473284

5 -2.998223 1.432301 0.891968

1 -2.830584 2.444227 1.473312

5 -2.141488 0.000013 1.467844

1 -1.356628 0.000014 2.342071

5 -2.141446 -0.000013 -1.467877

1 -1.356384 -0.000034 -2.341923

6 -0.352137 -1.448423 0.000052

6 -1.671045 -0.818525 0.000068

6 -1.671047 0.818516 0.000051

6 -0.352139 1.448418 0.000026

6 3.268287 0.733471 -0.000002

6 3.268288 -0.733471 0.000010

6 2.079429 -1.412486 0.000031

6 0.812719 -0.739107 0.000050

6 0.812719 0.739104 0.000038

6 2.079427 1.412485 0.000008

1 2.080465 2.497028 -0.000002

1 2.080468 -2.497029 0.000040

1 -0.337941 2.530856 -0.000013

6 4.539395 1.414517 -0.000024

6 5.704177 -0.720781 -0.000038

1 4.539053 -2.498712 0.000011

6 5.704177 0.720784 -0.000050

1 4.539050 2.498714 -0.000032

1 6.651617 -1.246322 -0.000061

1 6.651616 1.246326 -0.000084

anthraceneCB

1 5 0 -3.964432 -0.070752 1.425393

2 1 0 -4.586897 -0.156664 2.424333

3 5 0 -4.541171 0.789102 -0.026730

4 1 0 -5.598177 1.313193 -0.059943

5 5 0 -4.399474 -0.994350 -0.027504

6 1 0 -5.255045 -1.798280 -0.124611

7 6 0 -2.933338 -1.290406 -0.808427

8 1 0 -2.754835 -2.209780 -1.345526

9 5 0 -3.163227 1.435584 0.897107

10 1 0 -3.220968 2.436556 1.521640

11 5 0 -1.666352 0.986625 0.037452

12 1 0 -0.320697 -2.447461 0.014032

13 5 0 -3.122814 1.442163 -0.892893

14 1 0 -3.145953 2.432874 -1.534962

15 5 0 -3.905927 -0.047145 -1.448609

16 1 0 -4.413859 -0.233829 -2.495140

17 5 0 -2.204378 0.065290 1.475185

18 1 0 -1.496322 0.010471 2.414243

19 5 0 -2.958226 -1.427173 0.907244

20 1 0 -2.772993 -2.489433 1.377312

21 5 0 -2.145690 0.084007 -1.425635

22 1 0 -1.438436 -0.048765 -2.354418

23 6 0 -1.644761 -0.708339 0.048939

24 6 0 4.538347 -1.453895 -0.007558

25 1 0 2.046888 -2.451035 -0.005928

26 6 0 2.077710 -1.366597 -0.003769

27 6 0 0.881766 0.825473 0.009047

28 6 0 0.830782 -0.657513 0.001328

29 6 0 2.176902 1.446188 0.003359

30 1 0 -0.123817 2.671711 0.010696

31 6 0 -0.254843 1.593403 0.021772

32 1 0 2.212514 2.530448 0.007212

33 6 0 4.637421 1.371778 0.001753

34 6 0 -0.344396 -1.364373 0.003888

35 6 0 3.291719 -0.730142 -0.004009

36 6 0 3.345343 0.732777 -0.000093

37 6 0 5.779244 0.640079 -0.000855

38 1 0 4.672084 2.455575 0.006059

39 6 0 5.726934 -0.799773 -0.005877

40 1 0 4.502346 -2.537604 -0.010563

41 1 0 6.744416 1.132206 0.001276

42 1 0 6.655223 -1.358884 -0.007744

anthraceneBBo

1 5 0 -3.854178 0.011266 -1.520957

2 6 0 -2.171869 0.020762 -1.206359

3 5 0 -1.632369 -0.859459 0.183567

4 1 0 -1.521323 -0.074642 -2.062082

5 1 0 -0.102948 2.580843 0.148381

6 1 0 -4.195693 -0.120530 -2.638363

7 6 0 4.642716 -1.413865 -0.034903

8 6 0 0.902563 0.744917 0.058913

9 6 0 0.901136 -0.748697 0.053705

10 5 0 -1.665879 0.915375 0.147523

11 5 0 -2.363566 0.023821 1.532111

12 1 0 -1.813996 0.016937 2.575838

13 5 0 -4.127599 0.006933 1.331442

14 1 0 -4.853990 -0.019804 2.261560

15 5 0 -3.166690 -1.423109 0.893034

16 1 0 -3.184530 -2.499999 1.371745

17 6 0 -2.996227 -1.297080 -0.789526

18 1 0 -2.858708 -2.164562 -1.417030

19 5 0 -3.200219 1.457398 0.872463

20 1 0 -3.259446 2.473768 1.471327

21 5 0 -3.020286 1.447959 -0.892232

22 1 0 -2.863535 2.357148 -1.625606

23 5 0 -4.541281 0.889218 -0.166831

24 1 0 -5.552666 1.480628 -0.306879

25 5 0 -4.509010 -0.898734 -0.147663

26 1 0 -5.413414 -1.625504 -0.350816

27 6 0 -0.240133 1.503384 0.111586

28 1 0 2.185221 2.488975 0.039999

29 6 0 4.644567 1.414473 -0.019722

30 6 0 -0.246820 -1.502158 0.108697

31 6 0 3.371785 -0.732354 -0.006430

32 6 0 3.373900 0.732085 0.001007

33 6 0 2.183227 1.404053 0.031705

34 1 0 -0.124038 -2.580858 0.152240

35 1 0 2.185609 -2.491168 0.018496

36 6 0 2.180951 -1.406154 0.018968

37 1 0 4.642604 -2.498218 -0.039028

38 6 0 5.807848 -0.720234 -0.052732

39 1 0 4.643251 2.498822 -0.012278

40 6 0 5.809735 0.721666 -0.044854

41 1 0 6.755059 -1.246333 -0.071979

42 1 0 6.757650 1.246378 -0.058150

anthraceneBBp

1 5 0 -2.210154 -0.000343 -1.455397

2 1 0 -1.600641 -0.000614 -2.464793

3 5 0 -1.634176 -0.886911 0.000272

4 6 0 4.660588 1.412936 -0.000165

5 5 0 -3.075466 -1.442009 -0.893743

6 1 0 -3.239255 -2.450775 -1.480533

7 6 0 -4.370790 -0.820407 0.000131

8 1 0 -5.341122 -1.291274 0.000198

9 5 0 -1.634186 0.886952 -0.000181

10 6 0 0.917439 0.745478 -0.000095

11 5 0 -3.075560 1.442011 0.893694

12 1 0 -3.239445 2.450759 1.480487

13 5 0 -2.210265 0.000351 1.455424

14 1 0 -1.600881 0.000661 2.464899

15 5 0 -3.075527 -1.441592 0.894405

16 1 0 -3.239405 -2.450077 1.481653

17 5 0 -3.075486 1.441582 -0.894458

18 1 0 -3.239302 2.450079 -1.481703

19 5 0 -3.953023 -0.000348 -1.449323

20 1 0 -4.735528 -0.000600 -2.326955

21 5 0 -3.953134 0.000330 1.449198

22 1 0 -4.735716 0.000567 2.326762

23 6 0 -4.370801 0.820387 -0.000288

24 1 0 -5.341139 1.291240 -0.000415

25 1 0 -0.089223 -2.585044 0.000481

26 6 0 -0.229307 1.506699 -0.000261

27 6 0 2.196616 1.402692 -0.000176

28 6 0 3.390242 0.731334 -0.000078

29 6 0 3.390242 -0.731336 0.000119

30 6 0 2.196614 -1.402691 0.000218

31 1 0 2.200343 2.488004 -0.000330

32 1 0 -0.089202 2.585053 -0.000439

33 1 0 2.200338 -2.488004 0.000371

34 6 0 4.660586 -1.412939 0.000207

35 6 0 0.917439 -0.745474 0.000153

36 6 0 -0.229310 -1.506687 0.000324

37 1 0 4.659594 -2.497568 0.000350

38 6 0 5.827427 -0.720573 0.000115

39 1 0 4.659596 2.497565 -0.000307

40 6 0 5.827428 0.720569 -0.000074

41 1 0 6.774693 1.247058 -0.000142

42 1 0 6.774692 -1.247063 0.000181

phenanthreneCC

5 3.649606 -0.434658 1.442688

1 4.210693 -0.576095 2.471473

5 4.391722 0.301966 0.000048

1 5.507980 0.685897 0.000056

5 3.130763 1.173844 0.891290

1 3.213718 2.196271 1.472248

1 -3.868567 2.793198 -0.000152

1 0.794139 2.870638 0.000079

5 3.954431 -1.434458 -0.000020

1 4.756462 -2.300682 -0.000065

5 2.432407 -1.605227 -0.891376

1 2.021552 -2.546021 -1.471263

5 3.649591 -0.434541 -1.442633

1 4.210655 -0.575890 -2.471445

5 3.130735 1.173912 -0.891117

1 3.213678 2.196424 -1.471928

5 2.432428 -1.605312 0.891330

1 2.021598 -2.546124 1.471203

5 1.952657 -0.007482 1.467376

1 1.189938 0.184172 2.339848

5 1.952681 -0.007376 -1.467343

1 1.189761 0.184406 -2.339609

6 0.555941 1.814552 0.000089

6 1.696787 0.895503 0.000129

6 1.300484 -0.682823 0.000066

6 -0.136676 -0.978552 -0.000003

6 -3.508408 0.645846 -0.000111

6 -3.093546 2.034933 -0.000111

6 -1.796922 2.389997 -0.000019

6 -0.735710 1.401904 0.000043

6 -1.102277 -0.030381 0.000019

6 -2.538747 -0.384393 -0.000023

6 -2.985702 -1.713317 0.000041

1 -1.508579 3.434732 0.000006

1 -0.374560 -2.031992 -0.000079

6 -4.873354 0.319002 -0.000173

6 -4.338254 -2.022101 -0.000004

1 -2.274443 -2.528306 0.000156

1 -5.601619 1.122426 -0.000259

6 -5.291618 -1.001321 -0.000162

1 -4.651275 -3.059239 0.000020

1 -6.348217 -1.240244 -0.000186

phenanthreneCB

1 5 0 -3.692173 -0.515884 1.416088

2 1 0 -4.278888 -0.764569 2.409338

3 5 0 -4.452610 0.208906 -0.024197

4 1 0 -5.603644 0.468350 -0.054893

5 5 0 -3.896480 -1.489784 -0.053559

6 1 0 -4.537082 -2.471613 -0.168498

7 6 0 -2.398696 -1.416594 -0.830552

8 1 0 -2.005458 -2.258309 -1.380590

9 5 0 -3.270168 1.148454 0.916378

10 1 0 -3.565875 2.096204 1.556083

11 5 0 -1.706620 1.076626 0.057468

12 1 0 0.362461 -1.950718 -0.020404

13 5 0 -3.230321 1.195010 -0.872353

14 1 0 -3.488817 2.162311 -1.497973

15 5 0 -3.637338 -0.430580 -1.456687

16 1 0 -4.086482 -0.715728 -2.507826

17 5 0 -2.015900 0.035244 1.477523

18 1 0 -1.313521 0.133004 2.417094

19 5 0 -2.393704 -1.583522 0.880800

20 1 0 -1.959758 -2.578811 1.333480

21 5 0 -1.959532 0.116380 -1.423304

22 1 0 -1.238725 0.170060 -2.349281

23 6 0 -1.292177 -0.554710 0.039566

24 6 0 4.906936 0.222542 -0.028158

25 6 0 2.934218 -1.722196 0.024792

26 6 0 2.540919 -0.374715 0.006486

27 6 0 0.813126 1.492348 0.029190

28 6 0 1.116510 0.039473 0.010031

29 6 0 1.928371 2.422177 0.010177

30 1 0 -0.572865 3.060284 0.049590

31 6 0 -0.460345 1.979858 0.052430

32 1 0 1.686249 3.478777 0.016624

33 1 0 4.016813 2.738995 -0.030463

34 6 0 0.130280 -0.895808 -0.007269

35 6 0 3.557273 0.610473 -0.014782

36 6 0 3.209003 2.015945 -0.015214

37 6 0 4.271006 -2.091452 0.011668

38 1 0 2.192208 -2.508309 0.055453

39 1 0 5.669152 0.993596 -0.045220

40 6 0 5.268176 -1.113711 -0.017265

41 1 0 4.537291 -3.141512 0.027097

42 1 0 6.313576 -1.398039 -0.026998

phenanthreneBBo

1 5 0 -3.587515 -0.440360 -1.528600

2 6 0 -1.964158 -0.024287 -1.195983

3 5 0 -1.265979 -0.684938 0.241165

4 1 0 -1.287191 -0.002078 -2.035935

5 1 0 -0.545462 2.981263 0.071367

6 1 0 -3.865165 -0.698587 -2.641596

7 6 0 4.979009 0.288801 -0.051105

8 6 0 0.836569 1.419486 0.039644

9 6 0 1.189496 -0.032262 0.081829

10 5 0 -1.708638 1.019051 0.119019

11 5 0 -2.210316 0.054746 1.537728

12 1 0 -1.693488 0.222860 2.584605

13 5 0 -3.915650 -0.384103 1.317778

14 1 0 -4.637984 -0.539527 2.238474

15 5 0 -2.636639 -1.565946 0.959107

16 1 0 -2.412889 -2.595364 1.487958

17 6 0 -2.465526 -1.478896 -0.724394

18 1 0 -2.114786 -2.317183 -1.307127

19 5 0 -3.341319 1.224110 0.801381

20 1 0 -3.649434 2.223913 1.349354

21 5 0 -3.123501 1.178226 -0.960427

22 1 0 -3.167408 2.065587 -1.734703

23 5 0 -4.489664 0.313060 -0.225067

24 1 0 -5.607847 0.644406 -0.403958

25 5 0 -4.044061 -1.416199 -0.120700

26 1 0 -4.749909 -2.342451 -0.297082

27 6 0 -0.441143 1.899476 0.068054

28 1 0 1.651979 3.431203 -0.011930

29 1 0 4.001506 2.771983 -0.042165

30 6 0 0.234688 -0.998005 0.196185

31 6 0 3.617421 0.628389 -0.010707

32 6 0 3.219918 2.020621 -0.016753

33 6 0 1.926533 2.382432 -0.000041

34 1 0 0.553414 -2.028921 0.291704

35 6 0 3.074428 -1.720501 0.005621

36 6 0 2.633557 -0.388350 0.027752

37 1 0 2.358226 -2.530400 0.015821

38 6 0 4.423152 -2.044539 -0.035107

39 6 0 5.386812 -1.034556 -0.060681

40 1 0 5.713478 1.086289 -0.076639

41 1 0 4.723821 -3.085441 -0.049033

42 1 0 6.441027 -1.282656 -0.091921

phenanthreneBBp

1 5 0 -2.026658 0.001249 -1.455735

2 1 0 -1.431843 0.141260 -2.464034

3 5 0 -1.262187 -0.721435 0.000230

4 1 0 4.018500 2.772666 -0.000165

5 5 0 -2.535366 -1.600643 -0.892656

6 1 0 -2.462342 -2.620331 -1.478938

7 6 0 -3.937976 -1.292696 0.000328

8 1 0 -4.774368 -1.973784 0.000472

9 5 0 -1.669277 0.991711 -0.000168

10 6 0 0.854489 1.412884 -0.000208

11 5 0 -3.198722 1.206573 0.893966

12 1 0 -3.591027 2.150539 1.480181

13 5 0 -2.026671 0.001889 1.455842

14 1 0 -1.431893 0.142377 2.464097

15 5 0 -2.535369 -1.600249 0.893478

16 1 0 -2.462373 -2.619684 1.480204

17 5 0 -3.198712 1.206184 -0.894412

18 1 0 -3.591014 2.149902 -1.481027

19 5 0 -3.722341 -0.400394 -1.449482

20 1 0 -4.486228 -0.581656 -2.324806

21 5 0 -3.722353 -0.399762 1.449748

22 1 0 -4.486256 -0.580608 2.325142

23 6 0 -4.314679 0.303273 -0.000025

24 1 0 -5.367481 0.537810 -0.000071

25 1 0 0.580409 -2.041959 0.000443

26 6 0 -0.425012 1.897213 -0.000313

27 6 0 1.943184 2.376968 -0.000258

28 6 0 3.238442 2.018902 -0.000154

29 6 0 3.638567 0.628608 -0.000067

30 6 0 2.653931 -0.389179 -0.000054

31 1 0 1.667670 3.425905 -0.000358

32 1 0 -0.523613 2.980234 -0.000445

33 6 0 3.101866 -1.719895 -0.000086

34 6 0 5.002379 0.292652 -0.000037

35 6 0 1.207071 -0.035918 -0.000005

36 6 0 0.251364 -1.009402 0.000238

37 1 0 2.388580 -2.532152 -0.000144

38 6 0 4.451900 -2.040268 -0.000064

39 6 0 5.414841 -1.028779 -0.000040

40 1 0 5.734468 1.093058 -0.000034

41 1 0 4.754682 -3.080901 -0.000090

42 1 0 6.470212 -1.274476 -0.000023

**Cartesian coordinates of reference aromatic systems.**

C2B10H12

1 5 0 -1.455107 0.002268 0.850733

2 1 0 -2.329502 0.004057 1.636745

3 5 0 -1.449507 0.000939 -0.910534

4 1 0 -2.471405 0.001855 -1.500256

5 5 0 -0.892312 -1.436061 -0.025888

6 1 0 -1.478158 -2.444445 0.139657

7 5 0 0.888949 -1.438213 -0.023841

8 1 0 1.472904 -2.447470 0.142999

9 5 0 -0.888879 1.438216 -0.028070

10 1 0 -1.472528 2.448184 0.135498

11 5 0 0.892337 1.436084 -0.026464

12 1 0 1.478518 2.444316 0.138797

13 5 0 0.002724 0.888496 -1.460101

14 1 0 0.004715 1.529079 -2.451408

15 5 0 0.000578 -0.890943 -1.458697

16 1 0 0.001040 -1.533422 -2.448775

17 5 0 1.451258 -0.002658 -0.907195

18 1 0 2.474419 -0.003687 -1.494759

19 1 0 2.325774 -0.001611 1.641687

20 6 0 -0.002372 -0.810178 1.272425

21 6 0 -0.000430 0.812330 1.271160

22 5 0 1.453110 -0.000823 0.853738

23 1 0 -0.000943 1.289466 2.238976

24 1 0 -0.003773 -1.285753 2.240924

Benzene

1 6 0 -1.394065 0.000000 0.000000

2 6 0 -0.697054 1.207294 0.000000

3 6 0 0.697039 1.207267 0.000000

4 6 0 1.394092 0.000000 0.000000

5 6 0 0.697039 -1.207273 0.000000

6 6 0 -0.697054 -1.207288 0.000000

7 1 0 -1.239026 2.146000 0.000000

8 1 0 1.239051 2.145982 0.000000

9 1 0 2.478035 0.000008 0.000000

10 1 0 1.239041 -2.145990 0.000000

11 1 0 -1.239036 -2.145992 0.000000

12 1 0 -2.478039 -0.000008 0.000000

Naphthalene

1 6 0 0.000000 1.242379 1.400112

2 6 0 0.000000 2.428509 0.707268

3 6 0 0.000000 2.428509 -0.707268

4 6 0 0.000000 1.242379 -1.400112

5 6 0 0.000000 -2.428509 -0.707268

6 6 0 0.000000 -2.428509 0.707268

7 6 0 0.000000 -1.242379 1.400112

8 6 0 0.000000 0.000000 0.715413

9 6 0 0.000000 0.000000 -0.715413

10 6 0 0.000000 -1.242379 -1.400112

11 1 0 0.000000 3.370517 1.243315

12 1 0 0.000000 3.370517 -1.243315

13 1 0 0.000000 1.240370 -2.484838

14 1 0 0.000000 -3.370517 -1.243315

15 1 0 0.000000 -3.370517 1.243315

16 1 0 0.000000 -1.240370 2.484838

17 1 0 0.000000 -1.240370 -2.484838

18 1 0 0.000000 1.240370 2.484838

Anthracene

1 6 0 0.000000 0.000000 1.401530

2 6 0 0.000000 0.000000 -1.401530

3 6 0 0.000000 1.220522 -0.721084

4 6 0 0.000000 1.220522 0.721084

5 6 0 0.000000 -3.652254 0.712151

6 6 0 0.000000 -3.652254 -0.712151

7 6 0 0.000000 -2.473938 -1.404680

8 6 0 0.000000 -1.220522 -0.721084

9 6 0 0.000000 -1.220522 0.721084

10 6 0 0.000000 -2.473938 1.404680

11 6 0 0.000000 2.473938 -1.404680

12 6 0 0.000000 2.473938 1.404680

13 1 0 0.000000 -4.596300 1.244260

14 1 0 0.000000 -4.596300 -1.244260

15 1 0 0.000000 -2.472014 -2.489257

16 6 0 0.000000 3.652254 -0.712151

17 6 0 0.000000 3.652254 0.712151

18 1 0 0.000000 4.596300 -1.244260

19 1 0 0.000000 4.596300 1.244260

20 1 0 0.000000 0.000000 -2.486908

21 1 0 0.000000 2.472014 -2.489257

22 1 0 0.000000 2.472014 2.489257

23 1 0 0.000000 -2.472014 2.489257

24 1 0 0.000000 0.000000 2.486908

Phenathrene

1 6 0 0.000000 0.678299 2.091216

2 6 0 0.000000 0.726969 -0.378009

3 6 0 0.000000 1.419817 0.865314

4 6 0 0.000000 -0.678299 2.091216

5 6 0 0.000000 -3.552470 -0.297639

6 6 0 0.000000 -2.873414 -1.528047

7 6 0 0.000000 -1.493734 -1.563439

8 6 0 0.000000 -0.726969 -0.378009

9 6 0 0.000000 -1.419817 0.865314

10 6 0 0.000000 -2.831789 0.876696

11 6 0 0.000000 2.831789 0.876696

12 1 0 0.000000 -4.635785 -0.274792

13 1 0 0.000000 -3.434814 -2.455009

14 1 0 0.000000 -0.999295 -2.525481

15 6 0 0.000000 1.493734 -1.563439

16 1 0 0.000000 -3.343113 1.833254

17 6 0 0.000000 2.873414 -1.528047

18 6 0 0.000000 3.552470 -0.297639

19 1 0 0.000000 -1.229049 3.025472

20 1 0 0.000000 3.343113 1.833254

21 1 0 0.000000 0.999295 -2.525481

22 1 0 0.000000 3.434814 -2.455009

23 1 0 0.000000 4.635785 -0.274792

24 1 0 0.000000 1.229049 3.025472
